# Responses of Coastal Marine Microbiomes Exposed to Anthropogenic Dissolved Organic Carbon

**DOI:** 10.1021/acs.est.0c07262

**Published:** 2021-02-19

**Authors:** Elena Cerro-Gálvez, Jordi Dachs, Daniel Lundin, María-Carmen Fernández-Pinos, Marta Sebastián, Maria Vila-Costa

**Affiliations:** † Department of Environmental Chemistry, IDAEA-CSIC, Barcelona, Catalunya 08034, Spain; ‡ Centre for Ecology and Evolution in Microbial Model Systems, EEMiS, Linnaeus University, Kalmar 35195, Sweden; § Department of Marine Biology and Oceanography, ICM-CSIC, Barcelona, Catalunya 08003, Spain

**Keywords:** organic pollutants, seawater, metatranscriptomics, metagenomics, plasticity, PAH, OPE, alkanes, marine microbial communities

## Abstract

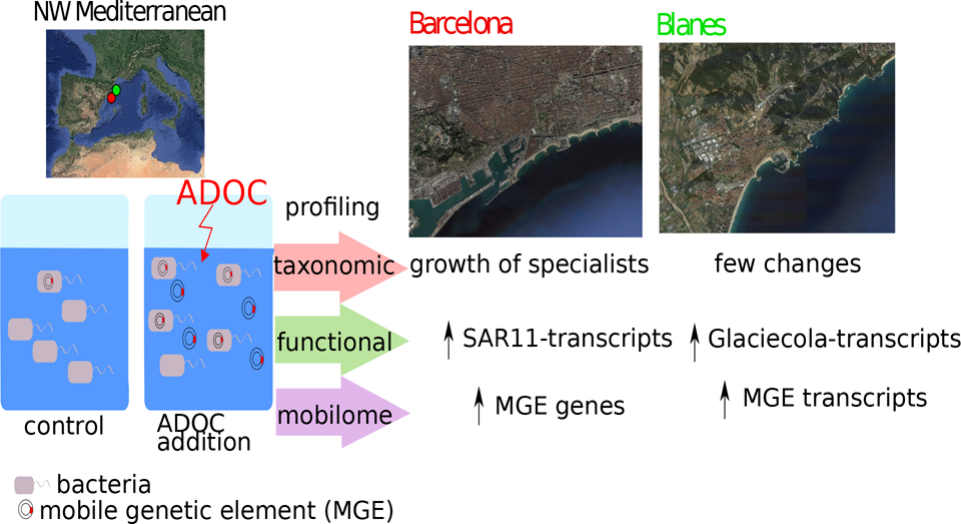

Coastal seawaters receive thousands of organic pollutants. However, we have little understanding of the response of microbiomes to this pool of anthropogenic dissolved organic carbon (ADOC). In this study, coastal microbial communities were challenged with ADOC at environmentally relevant concentrations. Experiments were performed at two Mediterranean sites with different impact by pollutants and nutrients: off the Barcelona harbor (“BCN”), and at the Blanes Bay (“BL”). ADOC additions stimulated prokaryotic leucine incorporation rates at both sites, indicating the use of ADOC as growth substrate. The percentage of “membrane-compromised” cells increased with increasing ADOC, indicating concurrent toxic effects of ADOC. Metagenomic analysis of the BCN community challenged with ADOC showed a significant growth of *Methylophaga* and other gammaproteobacterial taxa belonging to the rare biosphere. Gene expression profiles showed a taxon-dependent response, with significantly enrichments of transcripts from SAR11 and *Glaciecola spp.* in BCN and BL, respectively. Further, the relative abundance of transposon-related genes (in BCN) and transcripts (in BL) correlated with the number of differentially abundant genes (in BCN) and transcripts (in BLA), suggesting that microbial responses to pollution may be related to pre-exposure to pollutants, with transposons playing a role in adaptation to ADOC. Our results point to a taxon-specific response to low concentrations of ADOC that impact the functionality, structure and plasticity of the communities in coastal seawaters. This work contributes to address the influence of pollutants on microbiomes and their perturbation to ecosystem services and ocean health.

## Introduction

Chemical pollution is a poorly characterized vector of global change,^1^ especially under scenarios of chronic pollution such as the myriad of organic pollutants found at trace levels in the marine environment.^2−4^ Constraining this vector of global change is especially troublesome as most anthropogenic organic pollutants are unknown because of a lack of appropriate analytical procedures for their quantification.^5^ Anthropogenic dissolved organic carbon (ADOC) in coastal seawater originates from direct and indirect inputs of organic contaminants from rivers, effluents from wastewater treatment plants, continental runoff, groundwater, atmospheric deposition, and marine currents.^3,6^ The nonpolar (or hydrophobic) fraction of ADOC contains a large number of persistent chemicals that reach coastal waters, whereas many polar contaminants are at least partially biodegraded by microbiomes in wastewater treatment plants (WWTP) and during riverine transport.^7^ Hydrophobic ADOC derives from fossil fuels (mostly aliphatic and aromatic hydrocarbons), legacy persistent organic pollutants (POPs), organic pollutants of emerging concern (OPEC)^8^ and other anthropogenic chemicals. However, the myriad co-occurrences of hundreds of thousands of these anthropogenic chemicals at pico- and nanomolar concentrations can reach micromolar concentrations with still unquantified effects on ecosystems.^3,6^ Microbiome responses to ADOC include microbial degradation of some ADOC constituents under favorable conditions, but also a suite of antitoxic responses and adaptation strategies to ADOC.^9−12^ The use of some POPs is restricted by international legislation such as the Stockholm Convention due to their toxicity, persistence, potential for long-range transport, and bioaccumulation potential. Nevertheless, chronic pollution by ADOC remains uncharacterized in terms of Earth System functioning.^1^ The characterization of the effects of this pollution on marine microbial communities at environmentally relevant ADOC concentrations has received limited attention,^13,14^ even though microbial communities play a pivotal role driving the planetary biogeochemical cycles.

A deeper understanding of the pertinent traits of marine microbiomes facing realistic ADOC concentrations is hence long overdue and would provide invaluable information to assess ecosystem health in the form of insight into the extraordinary ability of microorganisms to adapt to small environmental changes thanks to the vast genetic pool they harbor.^15^ The challenge is discriminating which traits–among those related to taxonomy, functionality and physiology–can serve as biomarkers under realistic environmental settings. First, changes in the composition of marine microbiomes in response to specific ADOC compounds have been thoroughly analyzed under exposure to high concentrations, as these studies are usually performed under scenarios of oil spills and other accidental events needing bioremediation approaches. For instance, oil spill accidents promoted the growth of hydrocarbonoclastic bacteria, including members of the rare biosphere, pointing to the presence of a seed bank of bacteria that can bloom under hydrocarbon-rich conditions.^15^ However, the ecotoxicological assessment for acute events has limited applicability on the influence of ADOC on microbial communities under low levels of chronic marine pollution.^15,17−20^ Whether or not environmentally relevant concentrations of ADOC are selecting for populations in marine communities, and how the response is modulated by community fitness or other microbiome traits is still unclear for remote environments,^14^ and it has never been assessed in coastal waters from populated regions. Second, at the functional level, only a few genes involved in degradation of ADOC compounds have been identified, mainly those coding for hydrocarbon-degradation enzymes,^21^ limiting the use of genes as functional biomarkers for the overall ADOC pool.^22^ Previous exploratory work showed wide genomic responses to ADOC,^14^ but individual genes, as potential biomarkers, remain to be identified. Finally, at the physiological level, the impact of hydrophobic ADOC is generally focused on a few families of pollutants or oil extracts, representing only a partial view of ADOC effects. In terms of mass, the chromatographically unresolved complex mixture (UCM) is an important pool of the ADOC found in seawater,^4,23^ but it has received little attention in terms of effects to microbiomes.^14,24^ The handful of previous experiments that have challenged marine microbial communities with background concentrations of realistic ADOC mixtures, including the UCM, observed both biodegradation and a suite of physiological strategies against toxicity in heterotrophs and toxicological and inhibitory effects in phototrophs.^14,25^ These responses include those related to antioxidative stress and mechanisms to quench cell envelope stress, since hydrophobic ADOC strongly sorbs into membranes promoting harmful effects by narcosis,^24−26^ and other pollutant specific responses.^12,14^

Microbial responses to ADOC can be modulated by the history and environmental setting of the community. For example, pre-exposure of populations to low and high levels of contaminants modify the lag phase, rates, and extent of degradation.^10,27,28^ On the other hand, it has been shown that the adaptation of phytoplankton to organic pollutants can be modulated by dissolved organic carbon.^29^ Mobile genetic elements (MGE) promote genomic rearrangements and can take part in transfer of functional genes.^30^ In an environment exposed to pollutants, this can help spread genes involved in tolerance to pollutants, but also create genetic variation in the form of, for example, regulatory modifications, that, when modifying expression of genes involved in ADOC-response, can be picked up by selection.^27,28,31−35^ As high nutrient concentrations accelerate succession rates,^36^ these favor the proliferation of MGE within the communities^36−38^ potentially leading to an increase in evolvability through genomic rearrangements and gene transfer. The versatility of highly plastic marine microbiomes (with high abundances of MGEs) might result in higher potential for ADOC consumption and increased tolerance to ADOC compounds, since MGE represent a potent means of adaptation.^33,39−42^ However, few studies have addressed the synergistic effect of preadaptation and spread of MGEs for organic chemicals other than those dealing with antibiotics and their antibiotic resistance genes.^43−45^ Nevertheless, there is often the occurrence of multiple environmental stresses, for example, ADOC pollution at coastal sites is commonly linked to eutrophication,^46^ all of them driving microbial communities to higher plasticity potential.

The Mediterranean Sea is exposed to high anthropogenic pressures,^47^ but with considerable heterogeneity in their spatial distribution. The northwestern Mediterranean coast (Catalan coast, NE Iberian Peninsula) encompasses significant biological and physicochemical gradients including high variability in concentrations of micropollutants with higher levels closer to urban areas,^48−51^ and seasonal and spatial variations in nutrient limitation.^52,53^

In order to address the strategies to cope with hydrophobic ADOC within microbial communities in coastal systems, we performed experiments at two coastal sites from the NW Mediterranean with contrasting anthropogenic pressures in terms of pollutants and nutrients. The tested hypothesis was if microbial communities previously exposed to higher concentrations and more frequent pulses of organic contaminants and higher nutrient concentrations, show a higher plasticity that allow them to devote a higher percentage of their activity to consume these compounds than a less polluted and oligotrophic community. This was addressed by working at environmental relevant concentrations, analyzing the changes in microbial structure by metagenomics and changes in gene expression profiles by metatranscriptomic response (not only individual genes), and assessing the microbial response to the complex mixture of pollutants accounting for ADOC (not only individual pollutants).

## Methods

### Sampling Site Description

Experiments were performed with seawater collected off the Barcelona harbor (BCN) and at the Blanes Bay Microbial Observatory (BL) (Supporting Information (SI) Figure S1). BCN is representative of a polluted eutrophic coastal site (yearly average Chla >1.65 μg/L^54^). BCN receives freshwater inputs from the highly polluted Besòs and Llobregat rivers, runoff from the city of Barcelona, and there are tons of accumulated legacy organic pollutants in the sediment from past uncontrolled release of sewage sludge that can be resuspended during storm events. Approximately 60 km north from Barcelona, Blanes Bay (BL, NW Mediterranean) is representative of a rather oligotrophic coastal site moderately affected by human influences with low terrestrial inputs of nutrients (yearly average Chla <0.8 μg/L^55^).

### Concentration of ADOC and Preparation of ADOC Spike Solutions

With the objective to prepare the ADOC spike solutions to be used in the experiments, As much as 200 L of surface seawater was collected from BCN (26th May 2015; 41°22′16.4″N 2°11′23.3″E) and from a site located 50 km south of BCN (Vilanova i la Geltrú) with similar pollution levels than BL^50^ (6th June 2015; 41°06′48.0″N 1°47′38.4″E) using several 20 L metal carboys. Briefly, seawater was concentrated on a XAD-2 adsorbent and eluted with dichloromethane and methanol. After concentration, extracts were fractionated on an aluminum oxide (alumina) column using solvents of different polarity. Concentration of three characteristic and ubiquitous families of hydrophobic organic compounds belonging to ADOC (organophosphate esters (OPEs) flame retardants and plasticizers, polycyclic aromatic hydrocarbons (PAHs), and *n*-alkanes) were analyzed using the methods previously described.^4,12,56^ The 24 *n*-alkanes identified and quantified were a series from nC_12_ to nC_35_. The 64 target PAHs were naphthalene, methylnaphthalenes (sum of two isomers), dimethylnaphthalenes (sum of six isomers), trimethylnaphthalenes (sum of seven isomers), acenaphthylene, acenaphthene, fluorene, dibenzothiophene, methyldibenzothiophenes (sum of three isomers), dimethyldibenzothiopenes (sum of five isomers), phenanthrene, methylphenanthrenes (sum of four isomers), dimethylphenanthrenes (sum of seven isomers), fluoranthene, pyrene, methylpyrenes (sum of five isomers), dimethylpyrenes (sum of eight isomers), benzo[*g,h,i*]fluoranthene, benzo[*a*]anthracene, chrysene, methylchrysenes (sum of three isomers), benzo[*a*]pyrene, perylene, and dibenzo[*a,h*]anthracene. The 10 OPEs were triisobutyl phosphate (TiBP), tri-*n*-butyl phosphate (TnBP), tris(2-chloroethyl) phosphate (TCEP), tris(1-chloro-2-propyl) phosphate (TCPPs, 3 isomers), tris[2- chloro-1-(chloromethyl)ethyl] phosphate (TDCP), triphenyl phosphate (TPhP), 2-ethylhexyl diphenyl phosphate (EHDPP), and tris(2-ethylhexyl) phosphate (TEHP).The most apolar fractions, fraction 1 and 2 (F1 and F2), contain hydrophobic hydrocarbons and synthetic organic compounds with a large contribution of an anthropogenic UCM.^4,14,23^ Both fractions were merged as representative of ADOC, and used as spike solution in the experiments. This mixture of nonpolar ADOC is similar to that used in previous works.^14,24,57,58^

### Experiments with Natural Communities

Coastal seawater was collected from the surface (0.5 m depth) at a site close to Barcelona harbor mouth (16th June 2015, 41°22′16.4″N 2°11′23.3″E, “BCN”), as representative of a polluted eutrophic coastal site, and Blanes Bay (22nd June 2015, BL, 41°40′13.5″N 2°48′00.6″E, “BL”), as representative of a rather oligotrophic site with moderate pollution (SI Figure S1). Responses to ADOC additions were analyzed in two experiment types: dose–response and bacterial response. (1) *Dose–response experiment*: marine microbiomes were challenged with four different exposure concentrations of ADOC (1×, 7.5×, 40×, and 240× in situ concentrations). ADOC spike solution in acetone was added to 40 mL glass tubes (previously baked, 450 °C, 4 h) in the different treatments. The same volume of acetone with no ADOC was added to the controls. The acetone was let to evaporate under the hood for 2 h before seawater addition. To assess the importance of nutrient availability, we tested two different trophic conditions: an enrichment with P, N, and C (0.6 μM NaH_2_PO_4_·H_2_O, 2 μM (NH_4_)_2_SO_4_ and 24 μM glucose final concentration; “with nutrients”), to stimulate bacterial growth and prevent any potential nutrient limitation, and a control (“without nutrients”). Samples were incubated in duplicate in the dark at in situ temperature for 48 h. Treatments of the experiments were run in duplicates. Monitoring of the abundance of prokaryotic cells, leucine incorporation rates (as a proxy of bacterial production), the percentage of damaged or dead cells (NADS-), and the abundance of actively respiring bacteria (CTC+) were performed after 0, 4, 24, and 48 h of incubation (see below). (2) *Bacterial responses to ADOC:* A second set of experiments using the same seawater consisted on adding 7.5× ADOC concentrations dissolved in acetone to 2 L glass bottles (treatments), and only the solvent (acetone) to control bottles. The solvent was let to evaporate for 2 h before adding the seawater. The collected water was added to the glass bottles and incubated at in situ temperature and dark conditions for 24 h. The experiment was run in duplicate. We collected and preserved samples for molecular analyses as described below at 0.5 and 24 h. Additional samples were taken at the same time points to analyze prokaryotic abundance and production, and nutrient concentrations. The ADOC spike solution was generated from water of BCN and a location south of the BCN site, with similar pollution levels than BL^50^

### Biological Parameters and Their Significance

Bacterial community structure was characterized by bacterial abundance determined by flow cytometry (SI Text S1) and by sequencing community DNA by metagenomics. Bacterial community activities were determined by [3H] leucine incorporation rates (SI Text S1) as a proxy of bacterial production and by describing gene expression profiles by metatranscriptomics. General physiological characteristics of the microbial communities were characterized by nucleic-acid-double-staining (NADS) viability protocol that enumerate the cells with intact versus damaged membranes and by the 5-cyano-2,3-ditolyl tetrazolium chloride (CTC) protocol to quantify the abundance of highly respiring bacteria. Both NADS and CTC positive cells were quantified by flow cytometry. See details in the SI.^59−,62^

### Inorganic Nutrients Concentrations

Samples (10 mL) were kept frozen at −20 °C until analysis of dissolved inorganic nutrient concentration (nitrite (NO_2_^–^), nitrate (NO_3_^–^), ammonium (NH_4_^+^), and phosphate (PO_4_^3–^)) were done. Measurements were performed by continuous flow analysis (CFA) on a Bran+Luebbe following Hansen and Koroleff.^63^

### Nucleic Acids Extraction and Sequencing

After 0.5 and 24 h incubations of the second experiment, each bottle was filtered through a 3 μm pore-size 47 mm diameter polytetrafluoroethylene filter and bacterial cells were collected onto a 0.2 μm pore-size 47 mm polytetrafluoroethylene filter under low vacuum pressure. The duration of the filtration step was no longer than 15 min to minimize RNA degradation. Each filter was cut in two halves, one was placed in 1 mL RNAlater (Sigma-Aldrich, Saint Louis, MO) and the other one into 1 mL lysis buffer (50 mM Tris HCl, 40 mM EDTA, 0.75 M Sucrose) and stored at −80 °C to preserve RNA and DNA, respectively. DNA extraction for metagenomic analyses was performed following the protocol described elsewhere.^14^ To estimate absolute gene counts, we added a DNA standard (*Thermus thermophilus* DSM7039 [HB27] genomic DNA) that functioned as internal control at 0.5% of the total mass of extracted DNA. mRNA for metatranscriptomic analyses was extracted and amplified as described somewhere else^64^ with the modification of the use of mirVana isolation kit (Ambion) to extract the total RNA. Artificial mRNA was synthesized by in vitro transcription from a pGEM-3Z plasmid and used as internal standard at 0.5% final concentration in order to calculate absolute transcript abundances.^65,66^ Resulting DNA and amplified RNA were sequenced at the National Center for Genomic Analysis (CNAG, Barcelona, Spain) using Illumina high output mode HS200 2 × 100bp v4.

### Bioinformatics

DNA (metagenomics) and cDNA (metatranscriptomics) sequences were quality trimmed and internal standards and any remaining stable RNA was quantified and removed using the ERNE mapping program^67^ against the internal standard sequences and an in-house database of marine bacterial stable RNA sequences, respectively. Archaea-harbored reads were eliminated of the analyses because of very low abundance. Subsequently, read pairs were joined using the PEAR program (https://www.h-its.org/en/research/sco/software^68^). Joined pairs, as well as separate reads not corresponding to joined pairs were aligned to the NCBI RefSeq database (downloaded October 2016) using the Diamond aligner v0.8.25^69^ in blastx mode with default parameters. The resulting alignments were taxonomically and functionally classified with MEGAN 6.5.10^70^ and exported for further analysis in R/tidyverse.^71^ Search for specific transposases was performed with Pfam profiles, using HMMER.^72^ The list of the specific Pfam profiles used is listed in Brazelton and Baross.^73^ Metagenomic analyses resulted in a total of 341 million paired-end reads and an average of 28.4 million reads per sample in a typical length of 101bp. After quality-trimming and filtering of added internal standards, between 12.8 and 17.6% of the potential protein-coding reads were taxonomically annotated and between 32.5 and 47.9% were functionally annotated in SEED (SI Table S1). The sequencing of the metatranscriptomes resulted in a total of 949 million paired-end reads, 59.3 million raw reads per sample. After removal of rRNA, tRNA and internal standard reads, 369 million possible protein-encoding sequences remained, 23 million per sample. Among these, between 16.5 and 30.3 were taxonomically annotated and between 35.5 and 47.9 were successfully annotated to a SEED functional protein and category.

### Statistical Analyses

Data treatment and statistical analyses were performed with the R Statistical Software. Significant differences between treatments were tested with t-Student tests performed using the ‘t.test’ function and Tukey’s HSD posthoc test using the “TukeyHSD” with a threshold for the significance set at *p* < 0.05. ANOVA were carried out using the “aov” function. Principal component analysis (PCA) and Permutational multivariate analysis of variance (PERMANOVA) were carried out using the Vegan package with standardized data.^74^ Analysis of differential gene abundances and differentially expressed genes was performed with the “EdgeR” package.^75^ Counts were normalized by internal standard recoveries to get absolute counts as described elsewhere.^67^

## Results and Discussion

### Initial Characterization of Sampling Sites

#### Level of Pollution

Three model families of ubiquitous organic pollutants (64 PAHs, 24 *n*-alkanes and 10 OPEs) are surrogates of hydrophobic ADOC and were used to characterize the level of pollution at both sampling sites. On average, concentrations of dissolved PAHs, OPEs, and *n*-alkanes in BCN waters were 15-, 5- and 2-fold higher than those in “Vilanova i la Geltrú”, respectively, which water was used in BL experiments (SI Figure S1, Table S2). The higher level of pollution in BCN than in “Vilanova i la Geltrú” or BL is consistent with previous work in this region that identified the Barcelona’s metropolitan region as a source of organic pollutants to seawater sediments and waters.^48,56,76,77^ Indeed, the BCN site has been receiving large anthropogenic inputs of pollutants and nutrients from the impacted Besòs and Llobregat rivers, atmospheric inputs from adjacent urban and industrial regions, WWTP effluents, runoff from the city of Barcelona, high shipping traffic, and tons of accumulated legacy organic pollutants in the sediment from past uncontrolled release of sewage sludge that can be resuspended during storm events.^48,56,76,77^ Measured concentrations at both sites were within the order of magnitude of concentrations previously measured in the same region. Specifically, the apparently dissolved concentrations of *n*-alkanes in our study (n-C_12_–n-C_35_: 240 ng/L in BCN and 110 ng/L in “Vilanova i la Geltrú”, see SI Table S2 for details) were within the range of those reported in the NW Mediterranean Sea (n-C_15_–n-C_40_: 40–4600 ng/L,^12,78,79^) and in the Gulf of Gabès (SW Mediterranean) (n-C_15_–n-C_40_: 20–6300 ng/L^79^). The sum of PAH concentrations in the dissolved phase (∑_64_PAH: 1.2 ng/L in BCN and 0.078 ng/L in “Vilanova i la Geltrú”) were lower than those reported in coastal seawaters from the NW Mediterranean (∑_15_PAH: 3.6–30.7 ng/L,^79^), but within the same range as in the open NW Mediterranean (∑_19_PAH: 0.16–0.81 ng/L^56^). Among individual PAHs, phenanthrene was the most abundant compound (0.21 ng/L in BCN and 0.028 ng/L in “Vilanova i la Geltrú”) and the general profile was dominated by low molecular weight PAHs (2–4 rings), in agreement with previous studies.^12,56,81−83^ Concentrations of ∑_10_OPE (46 ng/L in BCN and 8.4 ng/L in “Vilanova I la Geltrú”) were in the range of those previously measured in NW Mediterranean Sea.^12,84^ Therefore, in the experiments, microbiomes were challenged to concentrations tipical of a large urban region (BCN) or coastal midsize cities representative of NW Mediterranean (“Vilanova i la Geltrú” and BL).

#### Level of Nutrients

Concentrations of nutrients were 8-fold higher in BCN than in BL (SI Table S3). Average concentrations of NO_2_^–^+NO_3_^–^ were 3.6 ± 0.01 μM in BCN, and 0.6 ± 0.4 μM in BL. NH_4_ concentrations were 8.2 ± 1.4 and 1.3 ± 0.5 μmol/L in BCN and BL, respectively, whereas PO_4_^3–^ concentrations were 0.5 ± 0.05 and 0.04 ± 0.00 μM. These nutrient concentrations indicate a higher degree of eutrophication in BCN than in BL waters as previously described.^54,55^

#### Characterization of the Microbiomes

Abundances of heterotrophic cells quantified by flow cytometry were 0.9- to 3.5-fold higher in BCN than in BL for both HNA and LNA cells (SI Table S4). At both sites, the heterotrophic bacterial community was dominated by HNA cells (77.7 ± 3.2% in BCN, 65.5 ± 5.9% in BL). Physiological traits of the communities indicated a higher proportion of active cells in BCN than in BL. Significantly higher leucine incorporation rates were observed in BCN than in BL (442.6 ± 27.1 pmol Leu/Lh and 36.28 ± 3.6 pmol Leu/L·h, respectively), as well as a larger percentage of highly respiring cells (CTC+) (6.5 ± 0.2% and 2.7 ± 0.2% in BCN and BL, respectively). On the other hand, the proportion of dead cells (as determined by the NADS protocol) was 1.2-fold greater in BCN than in BL. Significantly different composition was observed in both initial microbial communities according to metagenomic reads (PERMANOVA analyses, *R*^2^ = 0.82, *P* = 0.002). Proteobacteria was the dominant bacterial phylum in BCN and BL microbial communities, representing 68% and 76%, respectively. Bacteroidetes was the next most abundant group (22% in BCN and 11% in BL) at both sites. At the class level, Alphaproteobacteria was the most abundant (31% in BCN and 53% in BL), but the most abundant groups differed between locations at the order level. Rhodobacterales order was the most dominant Alphaproteobacteria (up to 12%) at BCN, whereas SAR11 accounted for a higher percentage (up to 31%) at BL. The contrasting relative abundance of the copiotrophs Rhodobacterales at BCN and the oligotrophs SAR11 at BL also reflects the different trophic status of the waters.^85^

Transposases were significantly more abundant in BCN than in BL metagenomes (0.05 ± 0.006% of total metagenomic reads in BCN and 0.03 ± 0.0005% in BL, *t* test, *P* < 0.05) (SI Figure S2). The higher abundances of MGEs are consistent with the BCN microbial community being exposed to anthropogenic stresses such as higher pollution and variable nutrient pulses.^30,40,41^ Similarly, highly impacted regions of the inner Baltic Sea have been shown to have higher abundances of transposases than in the adjacent marine waters.^42^

Thus, the concurrent high concentrations of pollutants, nutrients and MGEs agrees with a community in BCN adapted to episodes of high ADOC concentrations and nutrients variability, whereas in BL, waters were more oligotrophic and bacteria had a lower abundance of MGEs, consistent with a lower pre-exposure to anthropogenic stressors which presumably yielded a less resilient community.

### Effect of Nutrient Addition Without ADOC Additions

Nutrient addition (ammonium, dihydrogen phosphate and glucose) in the controls increased bacterial production 4.2-fold at BCN and 1.2-fold at BL after 48 h of incubation (SI Table S4). Significantly higher percentages of CTC-respiring cells were observed in nutrient amended controls at both sites after 48 h. Cell numbers of heterotrophic cells (LNA as well as HNA) doubled in the nutrient amended controls at both sites, and were especially high for LNA cells after 48 h (SI Table S4). The percentage of dead cells (NADS-) decreased during the incubation, showing a lower decrease in nutrient amendments at BCN (from 10.8 to 5.0%) than at BL (from 34 to 9.6%). These results show an overall stimulation of the community upon nutrient amendment at both sites in agreement with previous studies in NW Mediterranean coastal waters.^52,53^ Nutrient addition included addition of inorganic N and P forms plus labile organic C. The higher in situ concentrations of inorganic nutrients in BCN seawater than in BL and the higher increase of bacterial production in BCN than in BL seawaters and the similar increases of CTC-respiring cells at both sites, suggest a stronger limitation of labile C to build biomass in BCN microbiomes than in BL.

### Responses to ADOC with and without Nutrient Additions

**Figure 1:**
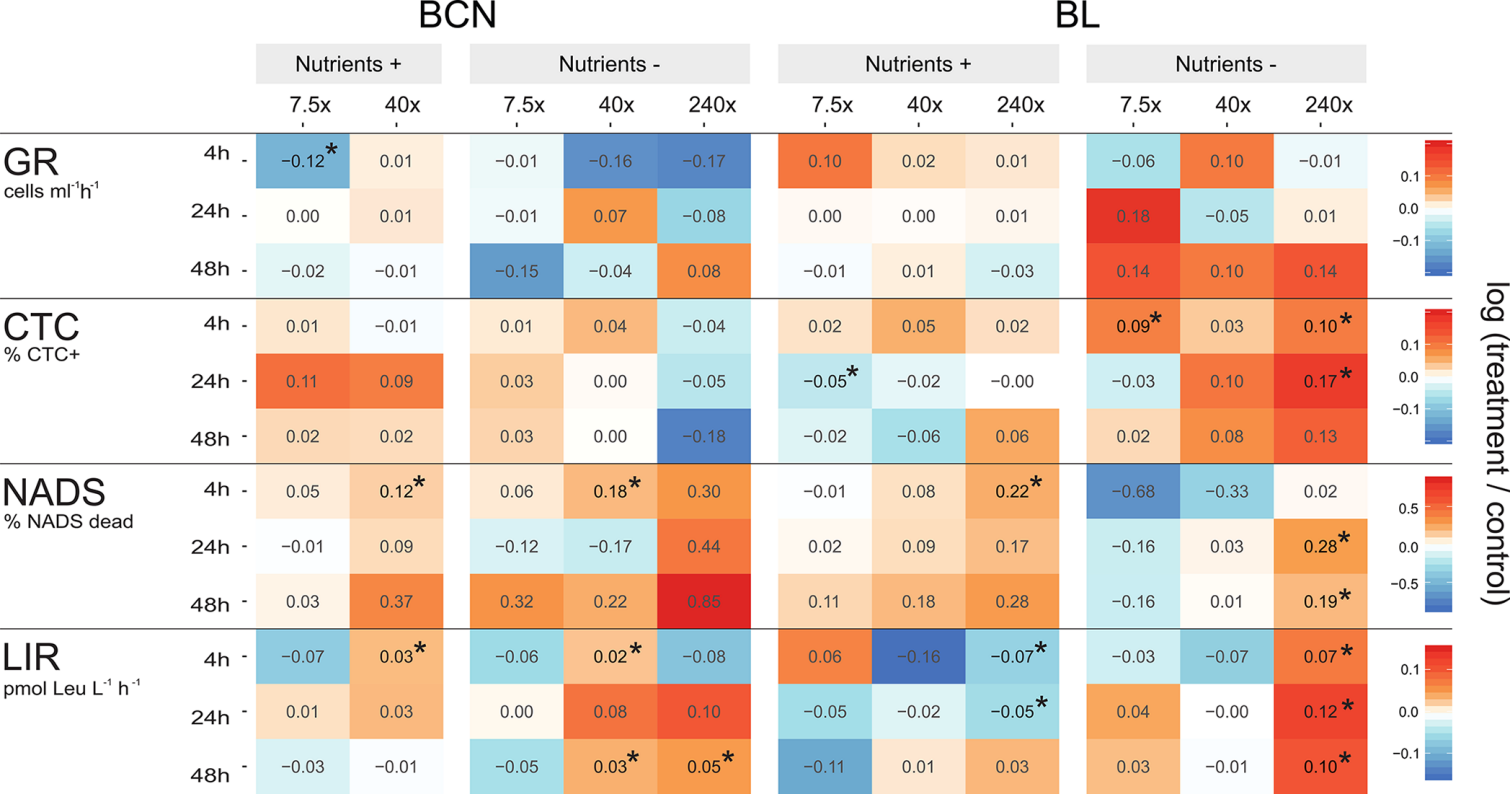
Heatmap of changes in biological measurements between controls and ADOC amendments (calculated as logarithm of change) in the dose-response experiments. Significant differences (*t* test; *P* < 0.05) between each treatment and control are marked with asterisks. GR: growth rates, LIR: leucine incorporation rates (bacterial production).

The dose–response experiment of ADOC treatments, with and without nutrient amendments, originated changes in physiological traits of the microbiomes, with changes more notable in the absence of nutrient additions (Figure 1). At BL, the percentage of highly respiring cells (CTC+) followed a similar trend as bacterial production, increasing steadily from low values similar to controls at 7.5× ADOC addition (7.4% of CTC+ cells), to higher numbers at 240× ADOC additions (12.7% of CTC+ cells) under ambient nutrient availability (Figure 1 and SI Figure S3). BCN communities did not show significant differences of CTC+ percentages between treatments and controls at any time or trophic condition. The percentage of CTC+ and LNA and HNA abundances, bacterial production and growth rates were positively correlated in the experiments (SI Figure S4). The percentage of dead cells (NADS-) relative to the total number of bacterial cells steadily increased with ADOC additions in BL, irrespective of nutrient availability (SI Figure S5). A similar pattern was observed in increases of NADS- along with ADOC amendments in BCN experiments, but with a lower magnitude. The percentage of dead cells increased, with a concurrent increase of biomass as shown by the positive correlation between dead cells and growth rates (*N* = 54, Pearson’s *r* = 0.49, *P* < 0.05; SI Figure S4). These results suggest that ADOC compounds were used as a substrate for growth by at least part of the microorganisms. As ADOC additions also led to an increased number of dead cells, especially in BL, ADOC produced a toxic effect on prokaryotes in addition to being a source of carbon and nutrients as previously observed in polar microbiomes.^14^ Community responses to ADOC under nutrient limiting conditions were then studied at gene level by means of metagenomic and metatranscriptomic approaches.

### Changes in Community Composition and Gene Content Due to Exposure to Background ADOC Concentrations

Metagenome responses to the effects of low concentrations of ADOC were studied after 0.5 and 24 h in incubations without nutrient additions. Treatments were done with a nominal increase of 7.5× ADOC compared to in situ concentrations. The nominal organic pollutant concentrations in the treatments fell in the range of 0.6–8.9 ng/L for ∑_32_PAH, 63–343 ng/L for ∑_10_OPE and 802–1767 ng/L for ∑_24_alkanes (n-C_12_–n-C_35_). These concentrations are similar to those previously measured in the dissolved phase of the Mediterranean Sea.^12,80,84,86^ Furthermore, for hydrophobic and semivolatile ADOC chemicals, it is known that real exposure concentrations are lower than nominal (spiked) concentrations due to adsorption to bottle walls, partitioning to cells, losses by volatilization, etc.^14,24^ Thus, cells were exposed to low ADOC concentrations, within the range of environmental variability.

**Figure 2:**
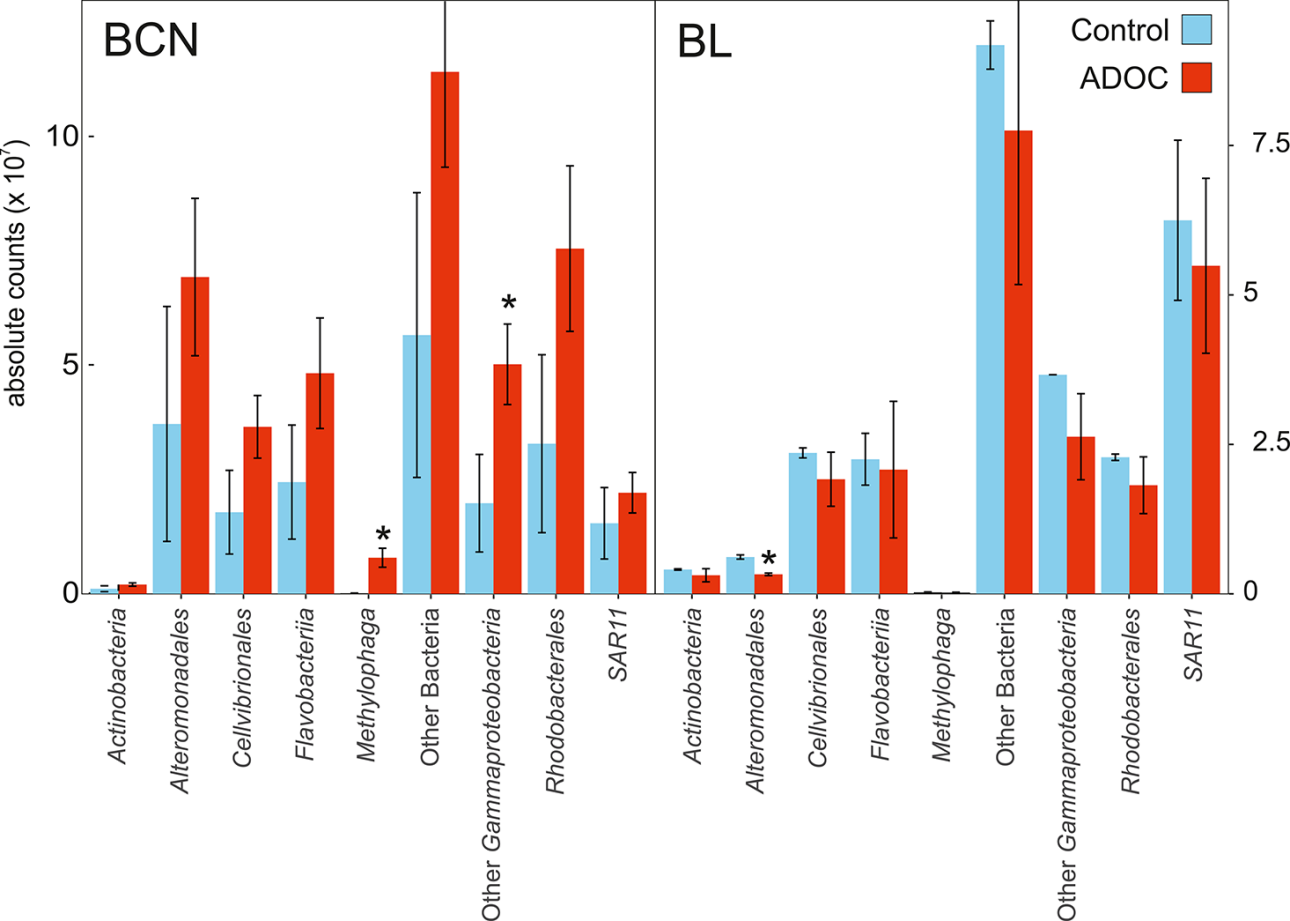
Taxonomical composition of Barcelona and Blanes metagenomes after 24 h of ADOC exposure. Asterisks indicate significant differences between ADOC amendments and controls (*t* test; *p* < 0.05). Values are means of duplicates. Error bars show standard deviation. ADOC: ADOC amendment.

**Figure 3:**
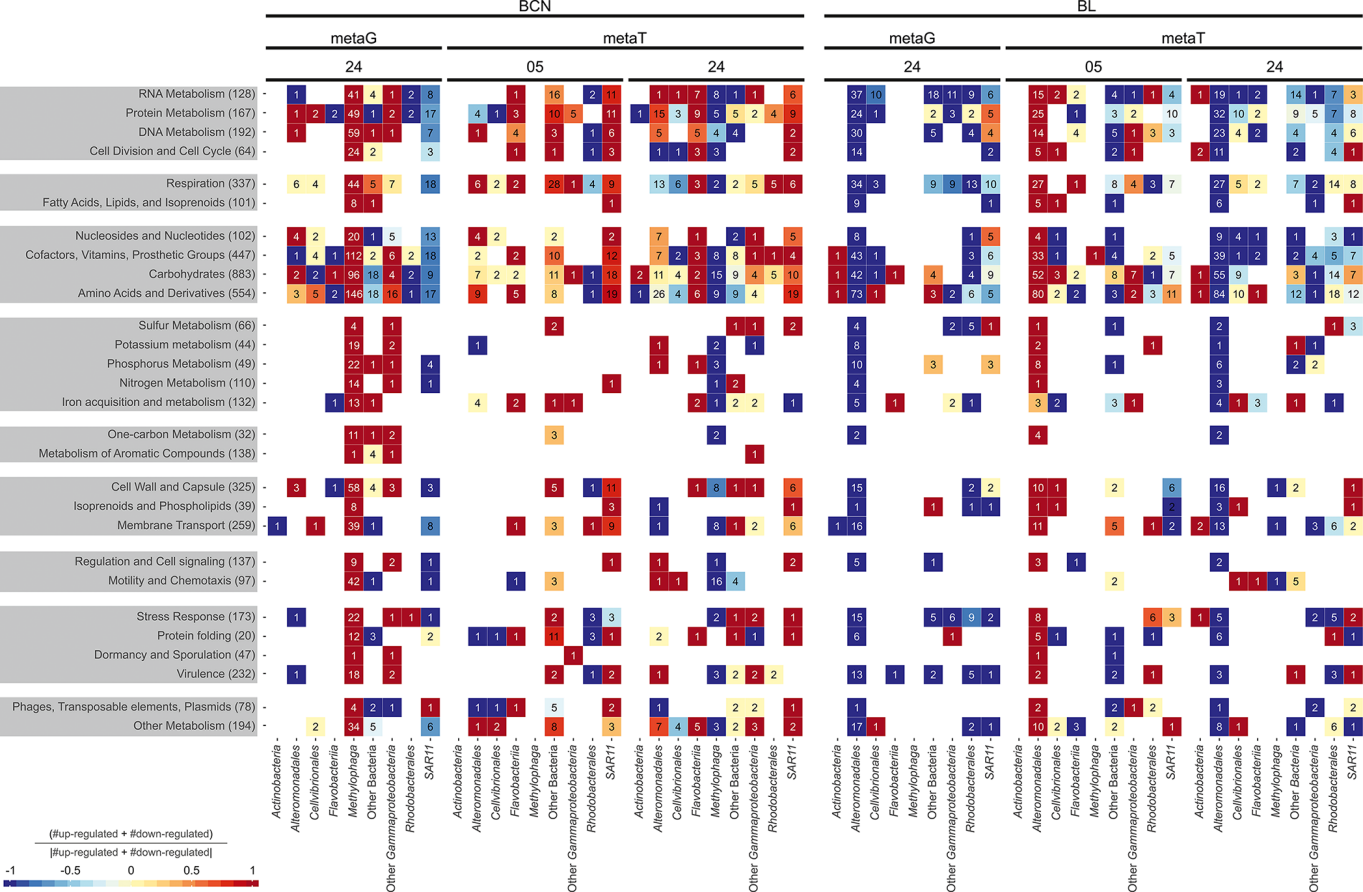
Total number of significantly enriched (in red) or depleted (in blue) genes and transcripts detected by edgeR (FDR < 0.05) in the experiments when comparing ADOC treatments and controls. Counts indicated inside each tile mean the number of genes with significant differences in that SEED category. Rows correspond to SEED categories and the value in parentheses is the total number of genes or transcripts that belong to that category.

**Figure 4:**
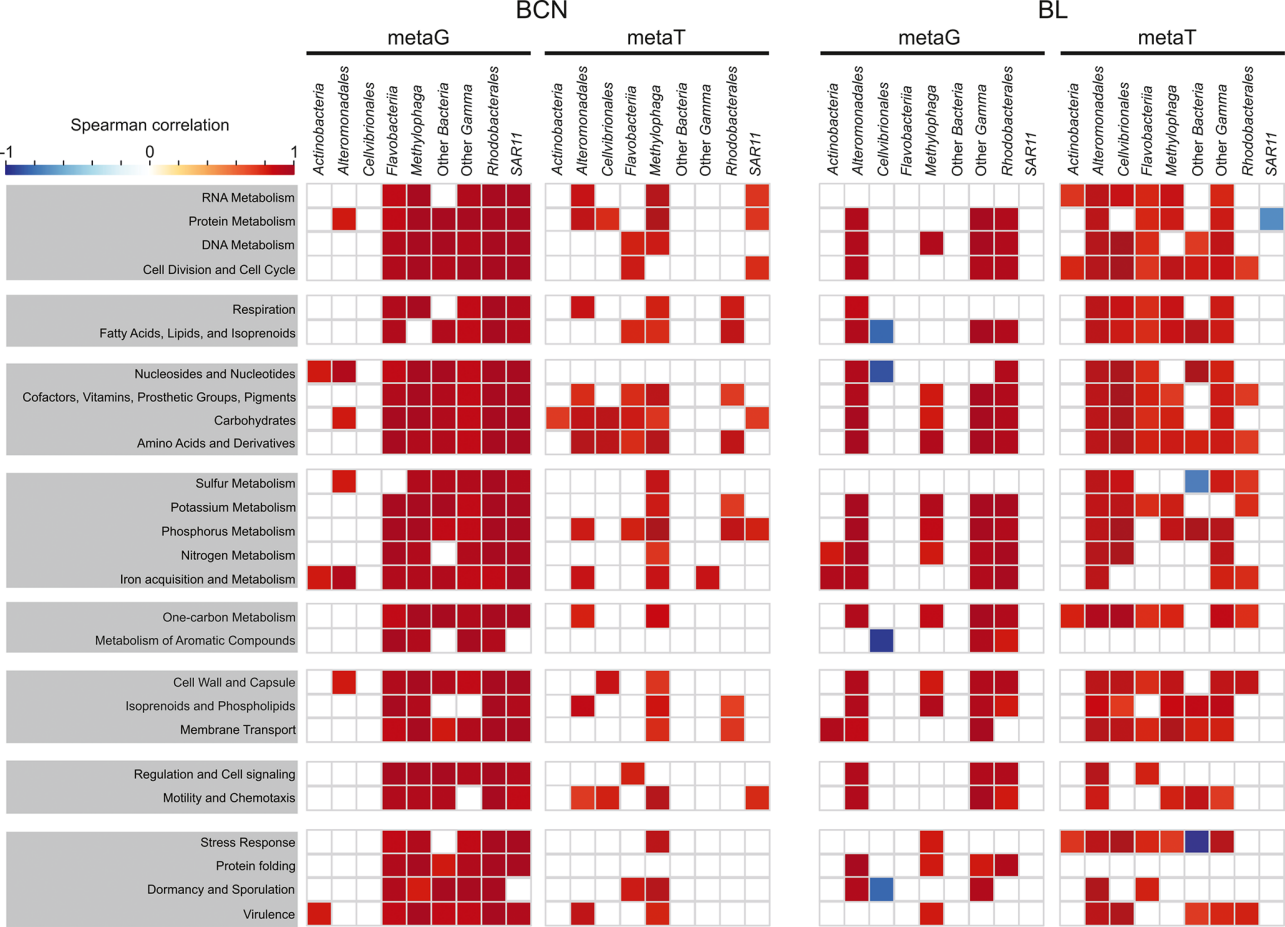
Spearman’s correlations between the relative abundance of transposons and the genes/transcripts that were significantly different between treatments and controls. Rows correspond to SEED categories.

ADOC addition promoted a notable change of the community composition in the BCN microbiome (SI Figure S6). Principal component analyses (PCA) indicated that Rhodobacterales and Gammaproteobacteria, especially the *Methylophaga* genus, were the most responsive bacteria to ADOC at the expense of SAR11 and Flavobacteriia (SI Figure S6). Significant (*p*-value <0.05) increases of *Methylophaga* and other Gammaproteobacteria populations were observed in ADOC treatments after 24 h (Figure 2). In BL, a significant decrease of Alteromonadales abundances was observed after 24 h between controls and ADOC treatments (Figure 2). At the functional level, after 24 h of incubation, different functional categories directly related to ADOC metabolism significantly increased in the BCN metagenomes, such as the metabolism of aromatic compounds and oxidative stress, mostly harbored by Rhodobacterales and Gammaproteobacteria (SI Table S5; Figure S7; *t* test, *p* < 0.05). Most of the significantly differentially abundant genes following ADOC incubations in BCN were harbored by *Methylophaga* and other Gammaproteobacteria (Figure 3) and were positively correlated with the relative abundance of transposases in the metagenomes (Figure 4), a trend observed in BL only for Alteromonas, other Gammaproteobacteria, and Rhodobacterales. Similarly, exposure of river biofilm to wastewater treatment plants (WWTP) waters has been shown to induce a concurrent dissemination of antibiotic resistance genes (ARGs) and MGEs.^45^ BL metagenomes showed a general decrease of significantly differentially abundant genes, especially those harbored by *Alteromonadales* (Figure 3).

**Figure 5:**
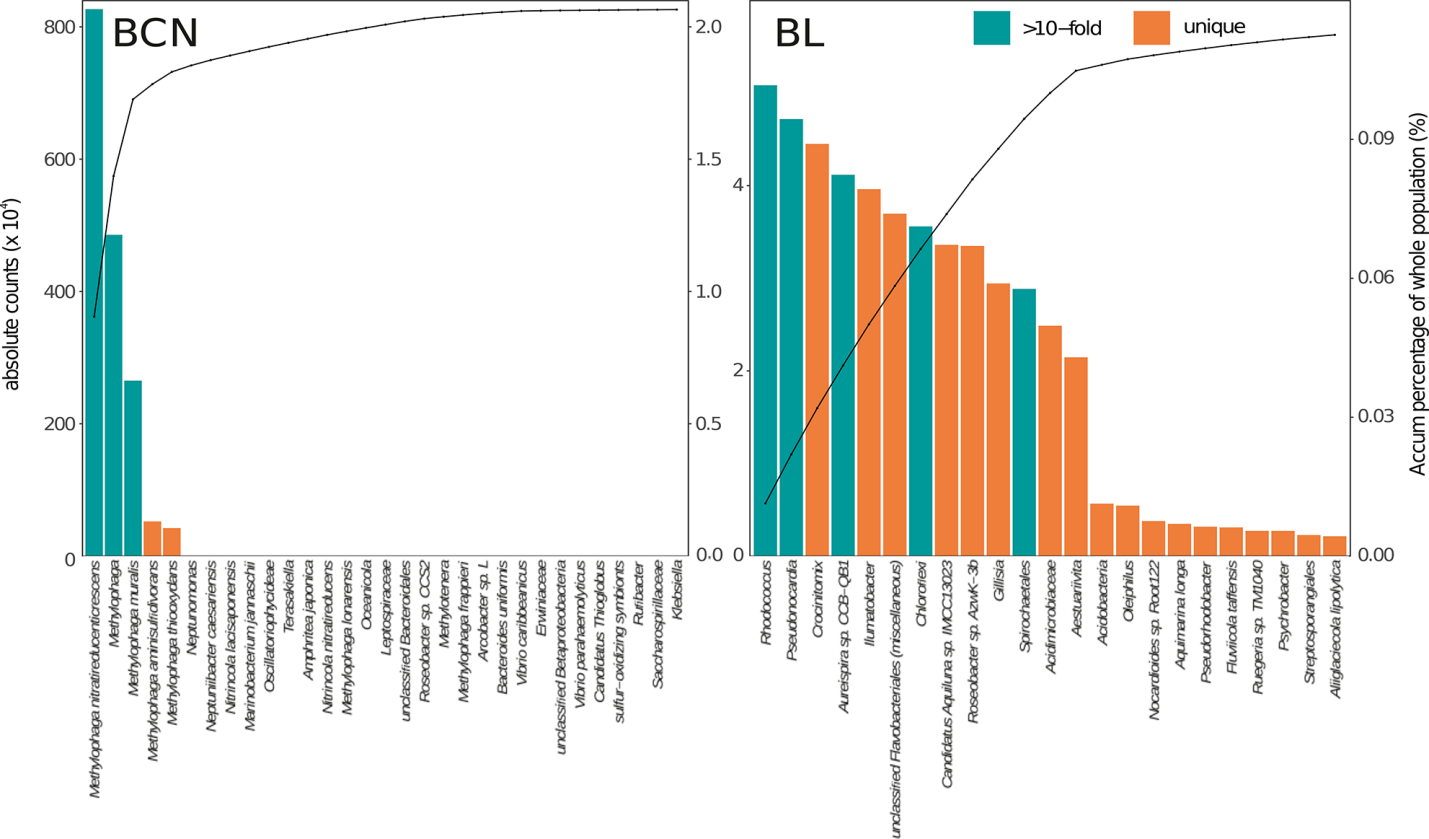
Growth of the rare biosphere. Abundances in the taxa that increased by 10-fold (green) or were absent in controls (orange) after 24 h of incubation in the Barcelona (BCN) and Blanes (BL) ADOC amendment. Phylotypes were taxonomically classified at highest resolution. Counts were summarized at each taxonomical level.

Growth of *Methylophaga* in BCN was especially relevant since they were at very low abundances in the initial community, constituting less than 0.03%, thus belonging to the rare biosphere. The taxa that increased in abundance more than 10-fold in ADOC treatments than in controls, or that were absent in controls but present in the treatments after 24 h, increased from 0.12% of total community in initial waters to 2.06% in ADOC treatments after 24 h (Figure 5). The fast ADOC-growing rare biosphere was mostly composed of *Methylophaga* species and other Gammaproteobacteria taxa in BCN (Figure 5). Specifically, *Methylophaga* spp. in the treatments, were 70-fold more abundant than in the controls. Similar increases were observed for other described ADOC-degrading genera such as *Alcanivorax* (2.1-fold increase), *Nocardioides* (2.1-fold increase), and *Pseudomonas* (2.3-fold increase) (SI Figure S8^21^). In BL, ADOC-stimulated taxa belonged to different groups, mostly Actinobacteria and Flavobacteriia, but their contribution to the total communities remained low (from 0.09% to 0.11% of total reads) after 24 h (Figure 5). Increases of specialized ADOC-consumers from the rare biosphere following ADOC pulses have been observed both at low concentrations in polar seawaters,^14^ and at high concentrations following oil spill accidents.^16,17,87^*Methylophaga* has been identified as an efficient hydrocarbon degrader in seawater^88^ and it can become enriched following oil spills.^16^ In the plume originated by the Deepwater Horizon oil spill in the Gulf of Mexico, methylotrophs in general were enriched in the community following increases of ADOC-degrading specialists.^89,90^ This can be explained by *Methylophaga* spp. having the capacity to remove methyl-groups from alkylated hydrocarbons, for example methyl phenanthrenes, that are generally abundant in the ocean^4^ and were abundant in the original ADOC (SI Table S2). This demethylation could be fast, and could thus have the potential to trigger an increase of methylotrophic transcripts after 24 h. Previous work has described methylotrophs after 24 h of exposure to ADOC in the Arctic^14^ and after several days when high MW DOC is degraded.^91^

### Gene Expression Profiles after ADOC Additions

Significantly enriched transcripts after 30 min and 24 h of ADOC addition were mostly related to heterotrophic activities of the cells, such as respiration, but also to effects on cells such as oxidative stress (Figure 3). Furthermore, an important number of transcripts related to cell wall and membrane transport and composition were enriched in the ADOC treatments, as previously observed in polar microbiomes.^14^ These transcriptomic responses are in agreement with the fact that hydrophobic ADOC accumulates in the membrane with concentrations many orders of magnitude higher than in seawater, inducing perturbation of cell membrane’s permeability, rigidity, and efficiency by inducing narcosis.^92,93^

All these responses were taxon-specific (Figure 3). The significant enrichment of transcripts related to antitoxicity strategies for SAR11 and Flavobacteriia in BCN and Alteromonadales, mostly the genus *Glaciecola,* in BL contrasted with a decrease of their relative abundance in the metagenomes after 24 h of ADOC incubation. In contrast, transcripts of the growing *Methylophaga* group could not be captured after 30 min and they were significantly depleted after 24 h. These trends can be explained by the coexistence in the communities of ADOC tolerant groups, ADOC-degrading taxa, groups negatively affected by ADOC, and by the different temporal responses to ADOC effects in the communities.^35,94^ The ADOC-degrading populations in a community can be tracked using genes and transcripts related to described degrading genes such as those included in the aromatic compounds SEED category. Although few of these transcripts were observed in the metatranscriptomes, significant enrichments of the aromatic compounds SEED category were observed in Rhodobacterales and some Gammaproteobacteria in ADOC challenged metagenomes (Figure 3 and SI Figure S7). In contrast, ADOC-tolerant communities may not grow on ADOC, but have a suite of antitoxicity strategies to cope with the pressure due to exposure to ADOC. These strategies, summarized for exposure to oil spills^95^ and to low concentrations of ADOC,^14^ allow them to compete with ADOC-degraders for the available nutrients. For example, as consumers of C1 chemicals, *Methylophaga* may not have the capacity to degrade ADOC compounds, but only the C1 compounds released by other bacteria. There were significant correlations between *Methylophaga*-harbored transposases and many significantly differentially abundant genes and transcripts between ADOC enrichments and the control, including those related to stress response, membrane transport, isoprenoids and cell walls^14^ (Figure 4). This suggests that the adaptation process in *Methylophaga* is at least partially related to MGE and methylotrophy (Figures 3 and 4). Another potential ADOC-tolerant community member was SAR11. SAR11 expressed most of the significantly differentially abundant transcripts in BCN microbiomes after 0.5 and 24 h, although did not significantly grow in cell number during the 24 h (Figure 3). A similar pattern for this group was observed in polar waters^14^ and was attributed to a higher tolerance to hydrophobic chemicals. For instance, SAR11 has a less hydrophobic cell surface compared to other taxa,^96^ which lowers the extent of adsorption of ADOC compounds and in turn lowers the risk of narcosis.

Alteromonadales accounted for most of the enriched transcripts after 0.5 h and most of the depleted ones after 24 h in BL transcriptomes (Figure 3). Most of the active Alteromonadales in BL corresponded to *Glaciecola* spp., a group that dominated the phenanthrene-tolerant community in NW Mediterranean coast, but not the phenanthrene-degrading community.^35^ These authors observed that *Glaciecola* accounted for most of the ^12^C-fraction after stable-isotope probing (SIP) incubations adding ^12^C- and ^13^C-phenanthrene in the less polluted sites (Marseille and Banyuls), with similar levels than BL bay. Our work agrees with these previous results and shows that *Glaciecola* cell activation can be observed at much lower ADOC concentrations (from mg/L in elsewhere^35^ to ng/L in our study).

### Do Pre-Exposed Microbiomes Show an Adaptation to Chronic ADOC Pollution?

The results obtained at the BCN and BL contrasting sites show a complex response to ADOC for two microbiomes with different previous exposure to ADOC and other environmental pressures. A surprising trend was the dissimilarity at both sites between metagenome and metatranscriptome responses to ADOC that could also be related to the different plasticity of BCN and BL microbiomes. Transposon genes were generally correlated with differentially abundant genes in BCN, but to a lower degree in BL. Conversely, transcripts of transposons were generally correlated with differential abundant transcripts in BL, but to a lower degree in BCN (Figure 4). As far as we know, this is the first time that transposons are measured using both metagenomes and metatranscriptomes under experimental conditions simulating an environmental stress (ADOC in this case). With the information available and current knowledge on the role of MGE it is not possible to provide a conclusive explanation to the patterns shown in Figure 4. We point here to a number of plausible explanations that will require further experimental validation such as (i) acquired plasticity versus developing plasticity, (ii) kinetic issues, (iii) trophic lifestyle of the community.

First, the results obtained suggest that potentially adapted communities, such as the ones from BCN, show transposons correlated with the genes responding to the stress. Conversely, in less adapted communities, such as in BL, the transcripts of transposons are generally correlated with the oxidative stress-related transcripts (Figure 4). The lack of significant changes of the bacterial community in BL, suggests that populations with the capacity to better withstand ADOC were lacking, or present at very low abundances, in the starting community, in contrast to the clearly more preadapted community in BCN that responded with differential growth of populations. During a longer time-span, ADOC-adapted populations may become detectable, and the increased expression of transposons that we observed in BL may have adaptive effects on the community. This may come in the form of horizontal transfer of ADOC-related genes associated with transposons, but possibly also as structural effects on genomes, that alter expression patterns. In BCN, pre-exposure to ADOC presumably originated a better adapted community to ADOC-induced stress. Transposons, like other MGEs, are often linked to fitness-related genes, in this case genes related to exposure to ADOC-defense genes. The transmission of these genes to a new genome likely increases the likelihood that the transposon will remain in the community.^97^ Evidence of linkages between MGEs and adaptive genes to organic pollutants have been observed in several bacterial isolates.^33^ One would thus expect to find more transposons in a community exposed recently to environmental stresses like pollution or antibiotics such in BCN to a greater extend than in BL. Previous studies have shown the concurrent dissemination (and correlation) of MGEs and tolerance genes such as antibiotic resistance genes (ARG).^98,99^ As far as we know, this is the first study that shows multiple correlations between MGEs (transposases) and the functions known to respond to stress, in this case exposure to ADOC. This observation will need further experimental validation.

Second, issues related to the time needed to respond to ADOC could also be behind the trends shown in Figure 4, as at both sites the metagenome and metatranscriptome were sampled after 24 h which could represent different stages of the microbial response to ADOC. Thus, another hypothesis is that Figure 4 mirrors a faster response for the more preadapted BCN population, but reflects the early stages of adaptation for the BL population. The influence of adaptation has received some attention in the terms of degradation processes. Pre-exposure of the BCN bacterial communities to ADOC and other anthropogenic pressures probably conferred to them a greater capacity to degrade ADOC compounds, as well as a higher transposons density (Figure 4 and SI Figure S2).

Third, the higher degree of eutrophication in BCN waters favored a higher abundance of copiotrophic bacteria. Copiotrophic bacteria harbor larger genomes than oligotrophic bacteria, with potentially higher number of transposons genes. However, while copiotroph genomes can be 20% larger than oligotrophs,^100^ the occurrence of MGE in copiotrophs versus oligotrophs has not yet been studied in marine bacteria. On the contrary, a recent work on soil bacteria chronology during forest recovery showed higher numbers of transposon genes in oligotrophic than copiotrophic bacteria,^101^ indicating that the abundance of these MGE depends mainly on the history of environmental changes (transitions between different environmental pressures) rather than on the level of environmental pressure (high nutrients). This counter-example suggests that the higher abundance of copiotrophs in BCN waters cannot explain their higher number of transposon genes, which may be related to a history of changing environmental pressures in terms of pollutants and nutrients. Furthermore, the higher abundance of copiotrophs in BCN does not explain the differences in ADOC responses in the metaT and metaG profiles in BCN and BL. In a previous work, coastal microbial communities in a eutrophic bay in the East China Sea under long-term WWTP’s effluent disturbances were less sensitive to inorganic nutrient input than a community free from direct anthropogenic disturbances.^102^

Future research will need to confirm the observations suggesting a potential relationship between MGE and responses to ADOC. As ADOC concentrations are increasing during the Anthropocene, this is an issue that will require further experimentation and an ambitious research agenda.

Refs (59, 60, 61, 62, 99)
