# Supplementary material for: Responses of Coastal Marine Microbiomes Exposed to Anthropogenic Dissolved Organic Carbon
Source: Environ Sci Technol. 2021 Feb 19;55(14):9609–21. doi: 10.1021/acs.est.0c07262 (PMC8491159; doi:10.1021/acs.est.0c07262)
Supplement: Supplementary file 1 — es0c07262_si_001.pdf [file es0c07262_si_001.pdf]

Supplementary Information

Responses of coastal marine microbiomes exposed to anthropogenic dissolved organic carbon

Elena Cerro-Gálvez[1], Jordi Dachs[1], Daniel Lundin[2], María-Carmen Fernández-Pinos[1], Marta Sebastian[3,4], Maria Vila-Costa[1]*

[1]Department of Environmental Chemistry, IDAEA-CSIC, Barcelona, Catalunya, Spain.

[2]Centre for Ecology and Evolution in Microbial Model Systems, EEMiS, Linnaeus University, Kalmar, Sweden.

[3]Department of Marine Biology and Oceanography, ICM-CSIC, Barcelona, Catalunya, Spain.

[4]Instituto de Oceanografía y Cambio Global, IOCAG, Universidad de Las Palmas de Gran Canaria, ULPGC, Spain.

*Corresponding author: maria.vila@idaea.csic.es

Supplementary Text, Tables and Figures captions

Text S1. Methods for flow cytometric determinations and bacterial production.

Table S1. Metagenome and metatranscriptome datasets from Barcelona (BCN) and Blanes (BL) experiments. C: Control; T: ADOC amendment; 0.5: time point after 30min; 24: time points after 24h; A and B: replicates.

Table S2. Seawater dissolved phase concentrations of n-alkanes, polycyclic aromatic hydrocarbons (PAH) and organophosphate esters (OPE) flame retardants and plasticizers in Barcelona (BCN) and Blanes (BL).

Table S3. Concentrations (in µmol/L) of inorganic nutrients in Barcelona (BCN) and Blanes (BL) experiments

Table S4. Biological parameters analyzed from the control bottles of dose-response experiment during the 48 h of incubation. Significant differences between control bottles with and without nutrients addition are represented with an asterisk (t-test; $P < 0.05$).

Table S5. Significant differences (t.test, $P < 0.05$) of the relative abundance of SEED categories between ADOC amendments (treatments) and controls in Barcelona (BCN) and Blanes (BL) experiments. Comparison column shows depletion or enrichments in the treatments versus the controls.

Figure S1: Location of the sampling site of the seawater used for the experiments in the Northwestern Mediterranean Sea. Bars indicate the pooled concentration of three families of organic pollutants (organophosphate esters (OPEs) flame retardants and plasticizers, polycyclic aromatic hydrocarbons (PAHs), and alkanes) measured in the dissolved phase at the surface (1 m).

Figure S2. Relative abundance of transposases in of Barcelona (BCN) and Blanes (BL) metagenomes (metaG) and metatranscriptomes (metaT) at initial time (05) and 24 h after exposure, in controls (C) and treatments (T). Values are means of duplicates. Error bars show standard deviation. ADOC: ADOC amendment.

Figure S3. Percentages of actively-respiring bacteria (CTC+) in four different exposure concentrations of ADOC (1x, 7.5x, 40x and 240x of *in situ* concentrations) with and without nutrient additions in the Barcelona (BCN) and Blanes (BL) experiments. Values are means of duplicates. Significant differences ($p < 0.05$) of mean values from replicates were analyzed using 1-way ANOVA followed by a post-hoc Tukey's HSD test and labeled as different letters.

Figure S4. Pearson correlations between different biological parameters analyzed in the dose-response experiments of ADOC treatments, with and without nutrient amendments, during the 48 h of incubation. GR: growth rates; HNA: high nucleic acid content; LNA: low nucleic acid content; BP: bacterial procudtion, %CTC+: Percentages of actively-respiring bacteria, %NADS dead : percentage of damaged or dead cells.

Figure S5. Percentages of damaged or dead cells (NADS-) in four different exposure concentrations of ADOC (1x, 7.5x, 40x and 240x of *in situ* concentrations) with and without nutrient additions in the Barcelona (BCN) and Blanes (BL) experiments. Values are means of duplicates. Significant differences ($p < 0.05$) of mean values from replicates were analyzed using 1-way ANOVA followed by a post-hoc Tukey's HSD test and labeled as different letters.

Figure S6. Principal component analysis (PCA) of the metagenomes in Barcelona (BCN) and Blanes (BL) using data from controls (C) and ADOC amendments (T) at initial time (0.5) and after 24 hours of ADOC exposure (24).

Figure S7. Relative abundance of genes grouped under SEED Metabolism of Aromatic compounds (top panel) and Stress Response (lower panel) in Barcelona (BCN) and Blanes (BL) metagenomes (metaG) after 24 h of ADOC exposure, in controls (C) and treatments (ADOC). Values are means of duplicates. Error bars show standard deviation. ADOC: ADOC amendment. Asterisks indicate significant differences between ADOC amendment and controls (t-test; $P < 0.05$).

Figure S8. Absolute abundances of specific hydrocarbonoclastic bacteria (taken from Ghosal et al 2016) in Barcelona (BCN) and Blanes (BL) metagenomes (metaG) after 24 h of ADOC exposure, in controls (C) and ADOC treatments (T).

References of the Supplemental material

Ghosal, D., Ghosh, S., Dutta, T.K., and Ahn, Y. (2016) Current state of knowledge in microbial degradation of polycyclic aromatic hydrocarbons (PAHs): A review. *Front. Microbiol.* **7**: 1369.

hydrocarbons (PAHs): A review. *Front. Microbiol.* **7**: 1369.

Text S1

Flow cytometric determinations. (1) *Prokaryotic cell abundance.* In order to quantify prokaryotic cell abundance, we collected duplicates of 1.8 ml from each bottle and fixed it with 1% buffered paraformaldehyde solution (pH 7.0) plus 0.05% glutaraldehyde, leaving it at room temperature in the dark for 10 min and stored it at -80°C. Prokaryotic cell abundance was later estimated by flow cytometry [59]. Heterotrophic prokaryotes cells were split into low and high nucleic acid content (LNA and HNA, respectively). Growth rates were calculated based on the slope of cell abundances versus time over the duration of the experiment. (2) *Abundance of membrane-compromised cells (NADS-).* The Nucleic-Acid-Double-Staining (NADS) viability protocol was used to enumerate the cells with intact versus damaged membranes [60]. NADS+ (assumed to be live, with intact membranes) and NADS- (assumed to be inactive, with compromised cell membranes, named as "dead") were counted by flow cytometry. (3) *Actively-respiring bacteria (CTC).* To quantify the abundance of highly respiring bacteria, we followed a protocol using 5-cyano-2,3-ditolyl tetrazolium chloride (CTC) described elsewhere [61]. Briefly, fresh sample aliquots (0.4 ml) received 5 mM CTC (from a fresh stock solution, 50 mM) and were incubated for 90 min in the dark at room temperature. CTC+ cells were enumerated by flow cytometry.

Bacterial production, by [^{3}H] Leucine incorporation rates . Heterotrophic bacterial production was estimated from [^{3}H] Leucine incorporation using the method described by Kirchman *et al.*[61], with the modifications suggested by Smith and Azam[62]. For each sample, triplicate aliquots (1.2 ml) and a trichloroacetic acid-killed control were incubated with 40 nM [^{3}H]Leucine for 1 h in the dark. Bacterial production is shown as [^{3}H]Leucine incorporation in pmol Leu/l·h.

Table S1. Metagenome and metatranscriptome datasets from Barcelona (BCN) and Blanes (BL) experiments. C: Control; T: ADOC amendment; 0.5: time point after 30min; 24: time points after 24h; A and B: replicates.

METAGENOMES_BCN

	C0.5A	C0.5B	C24A	C24B	T24A	T24B
Total joined reads	18,687,614	23,448,372	20,405,304	41,201,326	23,808,270	20,349,270
Internal standard reads	123,940	94,708	124,987	223,881	127,769	116,148
Standard normalization factor	36.9	30.1	54.8	11.4	76.4	69.8
Possible proteins	18,312,937	23,070,517	20,124,576	40,722,497	23,454,584	19,943,113
% potential protein-coding	98.0	98.4	98.6	98.8	98.5	98.0
SEED annotated proteins	2,781,887	3,655,843	3,408,756	6,598,355	4,127,096	3,417,074
% SEED annotated proteins	15.2	15.8	16.9	16.2	17.6	17.1
taxonomical annotated proteins	7,582,770	9,964,833	9,321,372	17,987,432	11,240,625	9,337,833
% taxonomical annotated proteins	41.4	43.2	46.3	44.2	47.9	46.8

METAGENOMES_BL

	C0.5A	C0.5B	C24A	C24B	T24A	T24B
Total joined reads	28,629,172	29,408,022	27,582,978	39,349,270	39,120,946	28,745,756
Internal standard reads	121,795	136,780	120,845	185,546	172,068	124,764
Standard normalization factor	11.0	12.8	38.8	25.9	16.2	37.9
Possible proteins	28,272,006	29,027,193	27,009,787	38,985,838	38,740,810	28,395,316
% potential protein-coding	98.8	98.7	97.9	99.1	99.0	98.8
SEED annotated proteins	3,627,340	4,136,437	3,985,255	6,259,450	5,567,487	4,290,304
% SEED annotated proteins	12.8	14.3	14.8	16.1	14.4	15.1
taxonomical annotated proteins	9,196,484	10,420,386	10,504,366	16,177,666	15,724,605	10,991,565
% taxonomical annotated proteins	32.5	35.9	38.9	41.5	40.6	38.7

METATRANSCRIPTOMES_BCN

	C0.5A	C0.5B	T0.5A	T0.5B	C24A	C24B	T24A	T24B
Total joined reads	71,196,214	65,749,050	80,443,490	59,187,424	45,460,596	49,141,324	72,055,396	69,498,944
Internal standard reads	3,333,633	1,733,772	2,855,576	1,836,766	1,715,675	1,402,204	2,081,764	2,063,560
Standard normalization factor	8,237	15,701	9,099	13,642	18,707	19,240	12,868	12,782
rRNA reads	38,549,874	35,077,320	41,627,924	30,912,998	23,877,182	26,128,926	40,196,870	36,769,092
% rRNA	54.1	53.4	51.7	52.2	52.5	53.2	55.8	52.9
Possible proteins	26,225,946	26,607,844	31,902,212	24,528,872	17,841,666	19,942,618	27,769,144	28,777,260
% potential protein-coding	36.8	40.5	39.7	41.4	39.2	40.6	38.5	41.4
SEED annotated proteins	5,829,599	7,034,102	6,727,065	7,427,642	4,051,560	5,409,757	6,567,605	7,661,793
% SEED annotated proteins	22.2	26.4	21.1	30.3	22.7	27.1	23.7	26.6
taxonomical annotated proteins	11,436,811	12,949,186	13,428,986	12,842,386	7,814,300	9,759,938	12,082,093	13,935,397
% taxonomical annotated proteins	43.6	48.7	42.1	52.4	43.8	48.9	43.5	48.4

METATRANSCRIPTOMES_BL

	C0.5A	C0.5B	T0.5A	T0.5B	C24A	C24B	T24A	T24B
Total joined reads	56,039,906	58,886,780	70,649,934	45,376,792	47,592,808	49,519,480	52,384,250	56,282,260
Internal standard reads	2,700,576	2,710,566	4,878,764	2,063,275	1,496,070	1,605,104	1,568,920	1,763,148
Standard normalization factor	2,352	2,447	1,649	3,796	7,362	6,339	6,653	7,338
rRNA reads	30,385,222	34,508,474	44,095,244	24,258,582	24,436,358	27,506,742	26,867,308	28,349,502
% rRNA	54.2	58.6	62.4	53.5	51.3	55.5	51.3	50.4
Possible proteins	21,211,720	20,412,994	19,970,666	17,250,244	20,105,176	19,286,648	22,540,612	24,482,494
% potential protein-coding	37.9	34.7	28.3	38.0	42.2	38.9	43.0	43.5
SEED annotated proteins	3,973,147	3,910,150	3,291,962	3,171,908	5,160,610	4,525,547	5,590,791	5,546,360
% SEED annotated proteins	18.7	19.2	16.5	18.4	25.7	23.5	24.8	22.7
taxonomical annotated proteins	8,309,550	8,027,193	7,637,533	6,579,092	9,637,850	8,563,266	10,427,887	10,773,189
% taxonomical annotated proteins	39.2	39.3	38.2	38.1	47.9	44.4	46.3	44.0

Table S2. Seawater dissolved phase concentrations of n-alkanes, polycyclic aromatic hydrocarbons (PAH) and organophosphate esters (OPE) flame retardants and plasticizers in Barcelona (BCN) and Blanes (BL).

		Concentration (ng/l)	
**Family**	**Compound**	**BCN**	**BL**
**n-alkanes**	Dodecane	0.148	< LOQ
	Tridecane	< LOQ	< LOQ
	Tetradecane	0.178	0.010
	Pentadecane	0.329	< LOQ
	Hexadecane	1.575	< LOQ
	Heptadecane	2.109	0.269
	Octadecane	3.527	0.558
	Nonadecane	1.282	0.207
	Eicosane	2.630	0.594
	Heneicosane	0.571	0.164
	Docosane	1.911	0.537
	Tricosane	1.182	0.301
	Tetracosane	2.596	0.935
	Pentacosane	2.164	0.862
	Hexacosane	4.735	2.043
	Heptacosane	9.043	2.438
	Octacosane	15.992	7.929
	Nonacosane	22.732	9.647
	Triacontane	29.232	13.629
	Hentriacontane	28.245	13.400
	Dotriacontane	29.664	14.347
	Tritriacontane	27.686	14.022
	Tetratriacontane	25.615	13.854
	Pentatriacontane	22.494	11.191
**PAHs**	Naphthalene	0.0125	0.0052
	Methylnaphthalenes	0.0020	< LOQ
	Dimethylnaphthalenes	0.0168	< LOQ
	Trimethylnaphthalenes	0.0531	0.0233
	Acenaphtylene	0.0040	< LOQ
	Acenaphtene	0.0032	0.0002
	Fluorene	0.0193	0.0031
	Dibenzothiophene	0.0074	0.0003
	Methyldibenzothiophenes	0.0072	0.0003
	Dimethyldibenzothiopenes	0.0302	< LOQ
	Phenanthrene	0.2116	0.0280
	Methylphenanthrenes	0.1147	0.0030
	Dimethylphenanthrenes	0.0993	0.0035
	Fluoranthene	0.2309	0.0050
	Pyrene	0.1405	0.0056
	Methylpyrenes	0.0232	< LOQ
	Dimethylpyrenes	0.0132	< LOQ
	Benzo[*ghi*]fluoranthene	0.0184	< LOQ
	Benzo[*a*]anthracene	0.0099	< LOQ
	Chrysene	0.0451	0.0001
	Methylchrysenes	0.0493	< LOQ
	Benzo[a]pyrene	0.0407	< LOQ
	Perylene	0.0337	< LOQ
	Dibenzo[*a,h*]anthracene	0.0018	< LOQ
**OPEs**	TiBP	1.705	0.287
	TnBP	1.346	0.171
	TCEP	2.780	0.479
	TCPP-1	13.866	2.705
	TCPP-2	11.684	2.506
	TCPP-3	11.932	1.659
	TDCP	1.336	0.289
	TPhP	0.097	< LOQ
	EHDPP	0.515	0.125
	TEHP	0.407	0.178

LOQ: Limit of quantification

Table S3. Concentrations (in µmol/L) of inorganic nutrients in Barcelona (BCN) and Blanes (BL) experiments.

		$NO_2^- + NO_3^-$	NH_4^+	PO_4^{3-}
BCN	C05	3.56 ± 0.01	8.21 ± 1.40	0.46 ± 0.05
	T05	4.99 ± 0.07	6.27 ± 1.17	0.23 ± 0.00
	C24	2.71 ± 0.79	4.00 ± 0.20	0.16 ± 0.00
	T24	2.95 ± 0.68	4.87 ± 0.74	0.17 ± 0.02
BL	C05	0.59 ± 0.42	1.26 ± 0.49	0.04 ± 0.00
	T05	0.42 ± 0.02	0.14 ± 0.10	0.05 ± 0.03
	C24	0.21 ± 0.05	0.61 ± 0.76	0.04 ± 0.01
	T24	0.14 ± 0.01	3.27 ± 1.15	0.03 ± 0.00

C: Control; T: ADOC amendment; 05: time point after 30min; 24: time points after 24 h; NO_2^-: nitrite; NO_3^-: nitrate; NH_4^+: ammonium; PO_4^{3-}: phosphate.

Table S4. Biological parameters analyzed from the control bottles of dose-response experiment during the 48 h of incubation. Significant differences between control bottles with and without nutrients addition are represented with an asterisk (t-test; $P < 0.05$).

			Nutrients -		Nutrients +		
		**timepoint (h)**	**mean**	**sd**	**mean**	**sd**	
BCN	GR	4	0.0362	0.0083	0.0322	0.0016	
		24	0.0354	0.0025	0.0847	0.0012	*
		48	0.0216	0.0115	0.0480	0.0010	
	HNA	0	3262185	81308	-	-	
		4	1148558	34836	1140472	11214	
		24	2453931	188628	8827115	223323	*
		48	2733586	1422849	5546308	169804	
	LNA	0	933558	74179	-	-	
		4	304996	13405	289609	2086	
		24	491990	10283	781805	42644	
		48	1076527	572745	7040535	778744	*
	% CTC+	0	6.5	0.2	-	-	
		4	6.5	0.2	7.0	0.3	
		24	5.3	1.7	26.5	9.4	
		48	6.9	3.0	30.2	4.6	*
	% NADS	0	10.7	0.9	-	-	
	dead	4	10.1	0.2	14.5	1.5	
		24	6.6	1.0	2.4	0.7	*
		48	3.9	0.3	13.3	11.7	
	LIR	0	442.6	27.1	-	-	
		4	1554.8	128.9	1767.7	22.3	
		24	970.8	142.0	3873.9	159.2	*
		48	1095.2	19.9	4575.0	234.6	*
BL	GR	4	0.0610	0.0024	0.0659	0.0014	
		24	0.0184	0.0020	0.0687	0.0030	*
		48	0.0256	0.0060	0.0503	0.0020	
	HNA	0	1272199	41954	-	-	

	4	322333	18503	337570	3721	
	24	602661	20945	2552133	203986	*
	48	1623625	355675	4323619	296142	*
LNA	0	669110	19205	-	-	
	4	354912	23005	353334	105	
	24	222355	61207	213513	3287	
	48	230139	175204	1620110	276950	*
% CTC+	0	2.7	0.2	-	-	
	4	3.3	1.0	3.4	0.1	
	24	9.1	0.9	56.2	0.2	*
	48	9.5	0.0	48.9	1.4	*
% NADS	0	8.9	2.0	-	-	
dead	4	7.5	1.4	34.1	1.8	*
	24	18.5	4.3	13.6	1.7	
	48	10.6	2.8	9.6	1.6	
LIR	0	36.3	3.6	-	-	
	4	34.8	10.1	34.0	5.6	
	24	946.9	241.8	1441.9	207.8	
	48	657.2	156.8	779.5	111.1	

GR: growth rates (cells/ml.h); HNA: high nucleic acid conten (cells/ml)t; LNA: low nucleic acid content (cells/ml); LIR: leucine incorporation rates as a measure of bacterial production (pmol leucine/L.h).

Table S5. Significant differences (t.test, $P < 0.05$) of the relative abundance of SEED categories between ADOC amendments (treatments) and controls in Barcelona (BCN) and Blanes (BL) experiments. Comparison column shows depletion or enrichments in the treatments versus the controls.

Location	Material	SEED category	*P* value	Comparison
BCN	metaG	Cell division and cell cycle	0.008	depleted
BCN	metaG	Cofactors, Vitamins, Prosthetic Groups	0.038	depleted
BCN	metaG	Metabolism of aromatic compounds	0.012	enriched
BCN	metaG	Nitrogen metabolism	0.019	enriched
BCN	metaG	Potassium metabolism	0.007	enriched
BCN	metaG	Stress response	0.023	enriched
BCN	metaT	Respiration	0.036	enriched
BCN	metaT	Unclassified	0.017	depleted
BL	metaT	Sulfur metabolism	0.025	depleted
BL	metaG	Nucleotide sugars	0.039	enriched

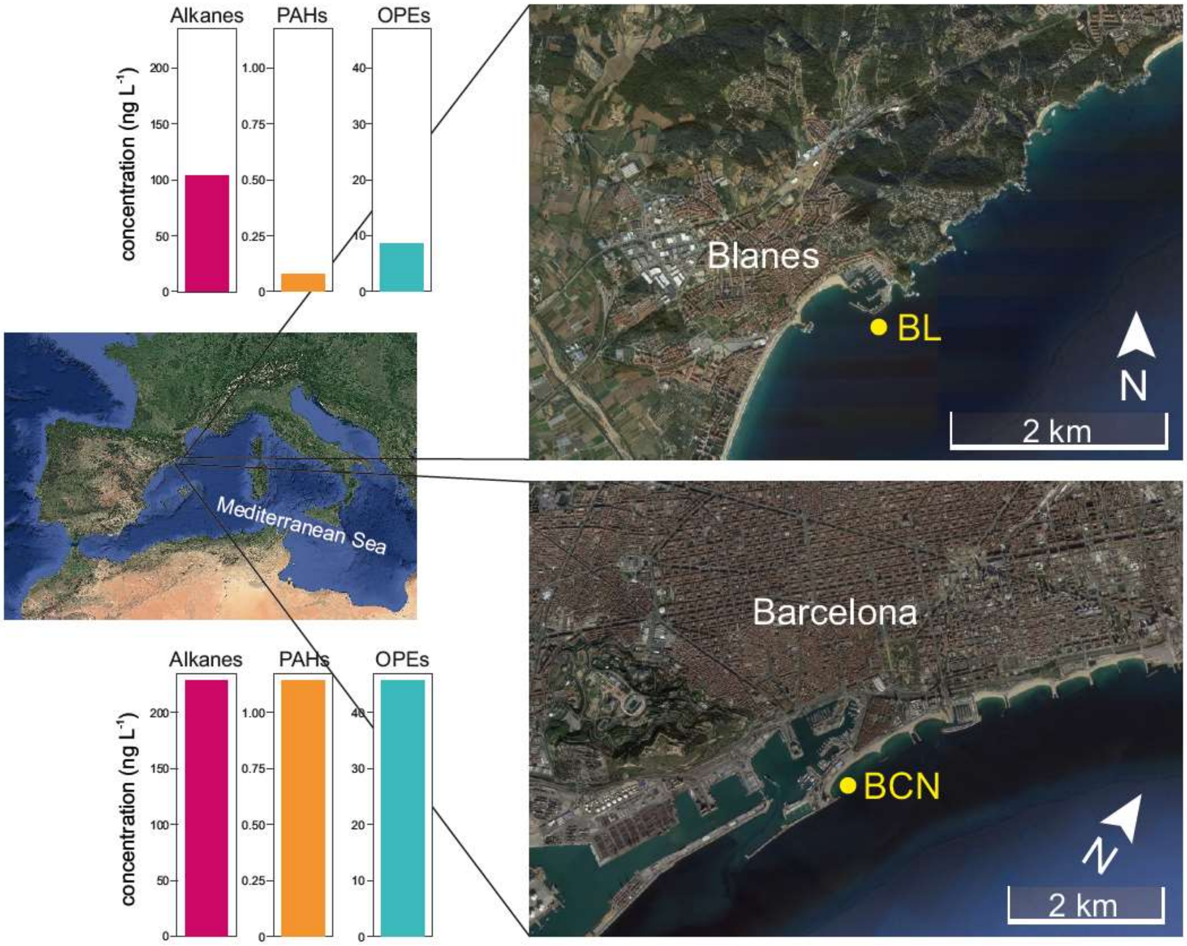

Figure S1: Location of the sampling site of the seawater used for the experiments in the Northwestern Mediterranean Sea. Bars indicate the pooled concentration of three families of organic pollutants (organophosphate esters (OPEs) flame retardants and plasticizers, polycyclic aromatic hydrocarbons (PAHs), and alkanes) measured in the dissolved phase at the surface (1 m) used to generate the ADOC mixtures spiked in the treatments at BCN and BL. See details in Table S2.

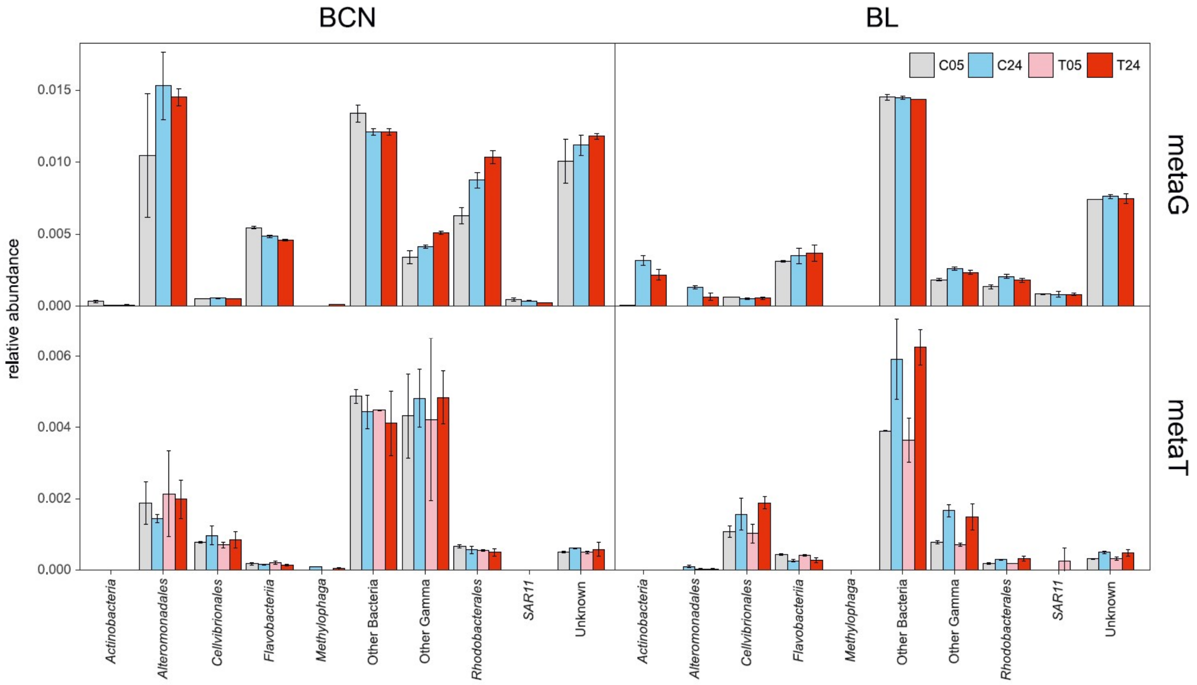

Figure S2. Relative abundance of transposases in of Barcelona (BCN) and Blanes (BL) metagenomes (metaG) and metatranscriptomes (metaT) at initial time (05) and 24 h after exposure, in controls (C) and treatments (T). Values are means of duplicates. Error bars show standard deviation. ADOC: ADOC amendment.

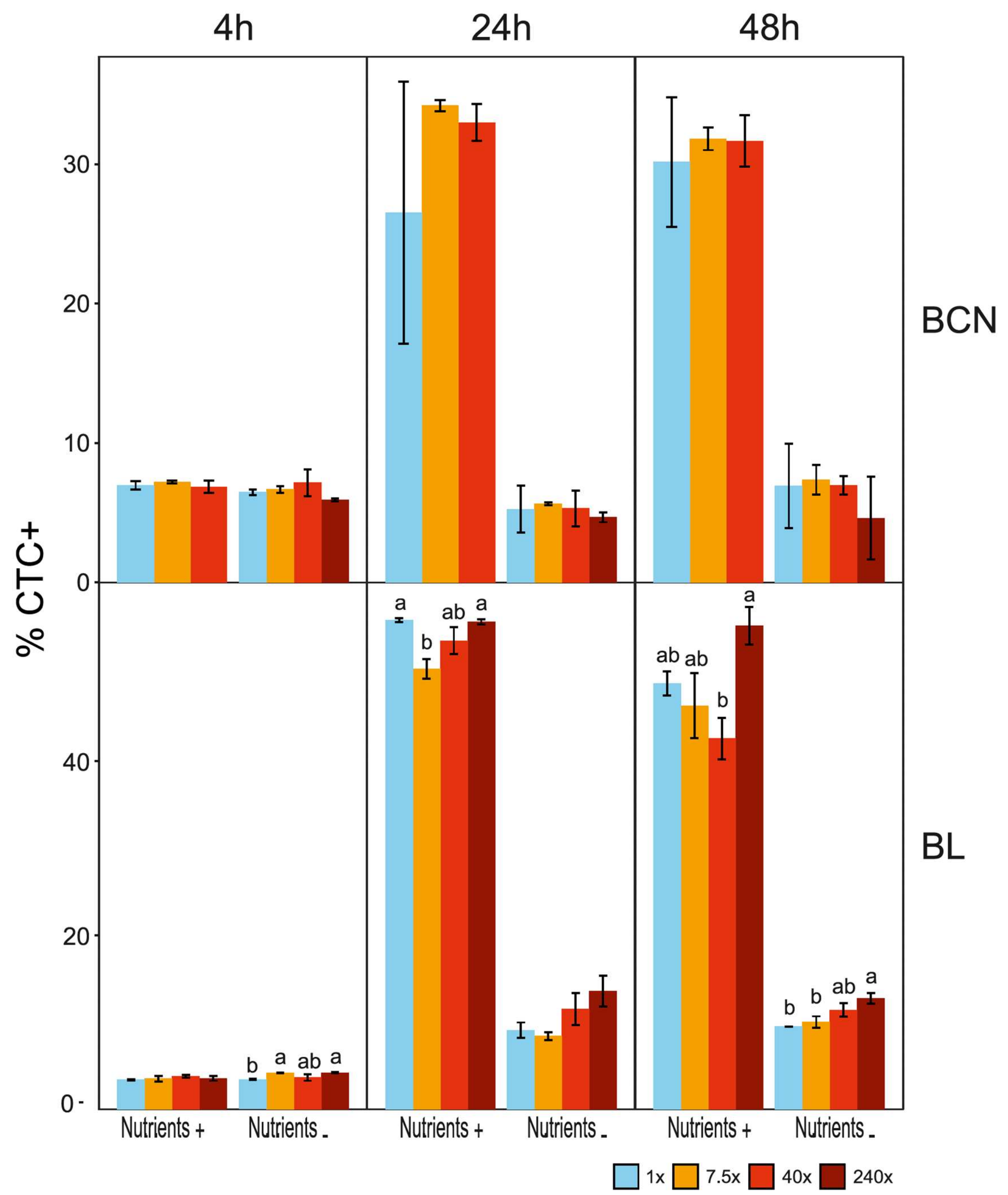

Figure S3. Percentages of actively-respiring bacteria (CTC+) in four different exposure concentrations of ADOC (1x, 7.5x, 40x and 240x of *in situ* concentrations) with and without nutrient additions in the Barcelona (BCN) and Blanes (BL) experiments. Values are means of duplicates. Significant differences ($p < 0.05$) of mean values from replicates were analyzed using 1-way ANOVA followed by a post-hoc Tukey's HSD test and labeled as different letters.

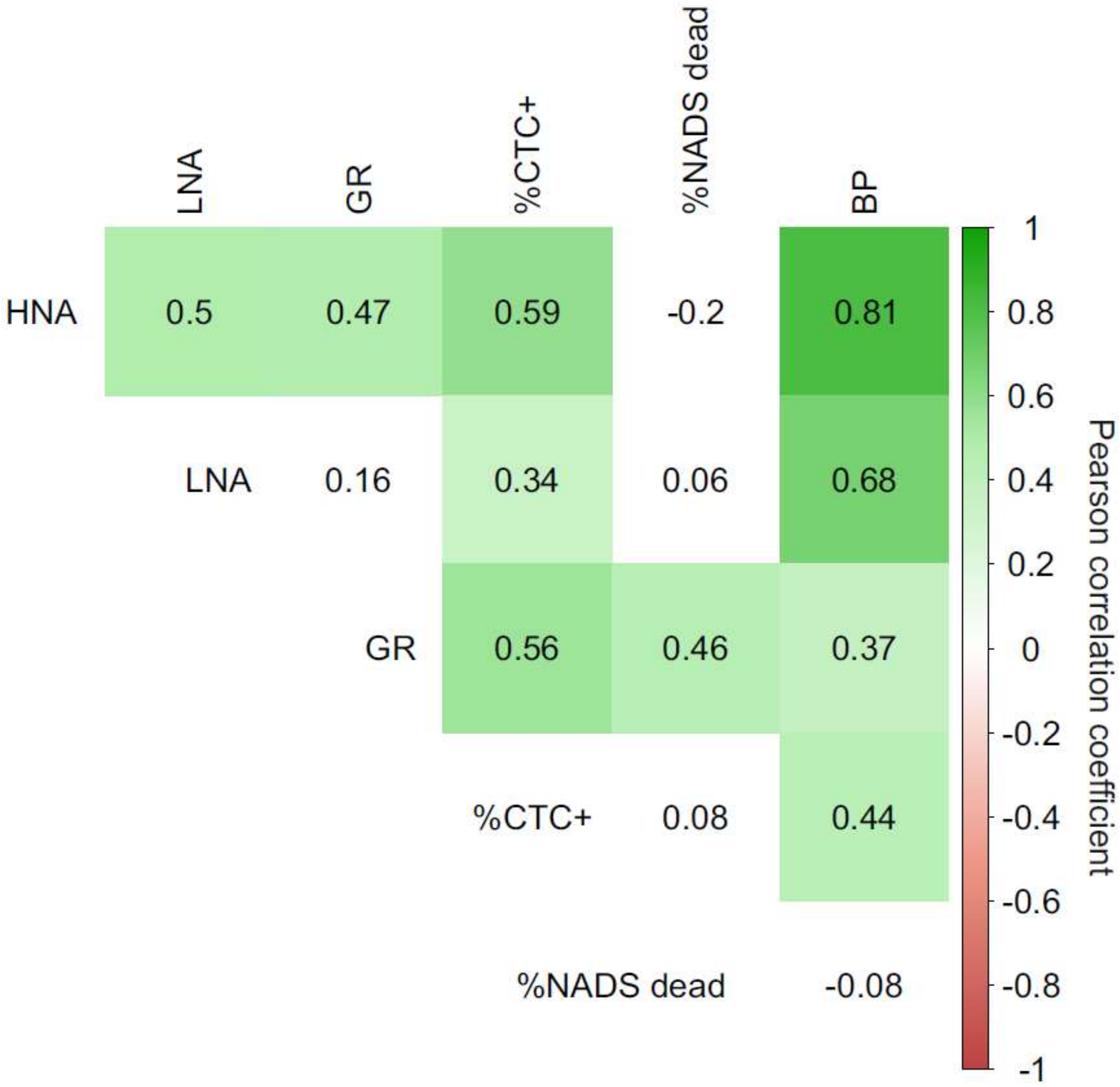

Figure S4. Pearson correlations between different biological parameters analyzed in the dose-response experiments of ADOC treatments, with and without nutrient amendments, during the 48 h of incubation. GR: growth rates; HNA: high nucleic acid content; LNA: low nucleic acid content; BP: bacterial procudtion, %CTC+: Percentages of actively-respiring bacteria, %NADS dead: percentage of damaged or dead cells.

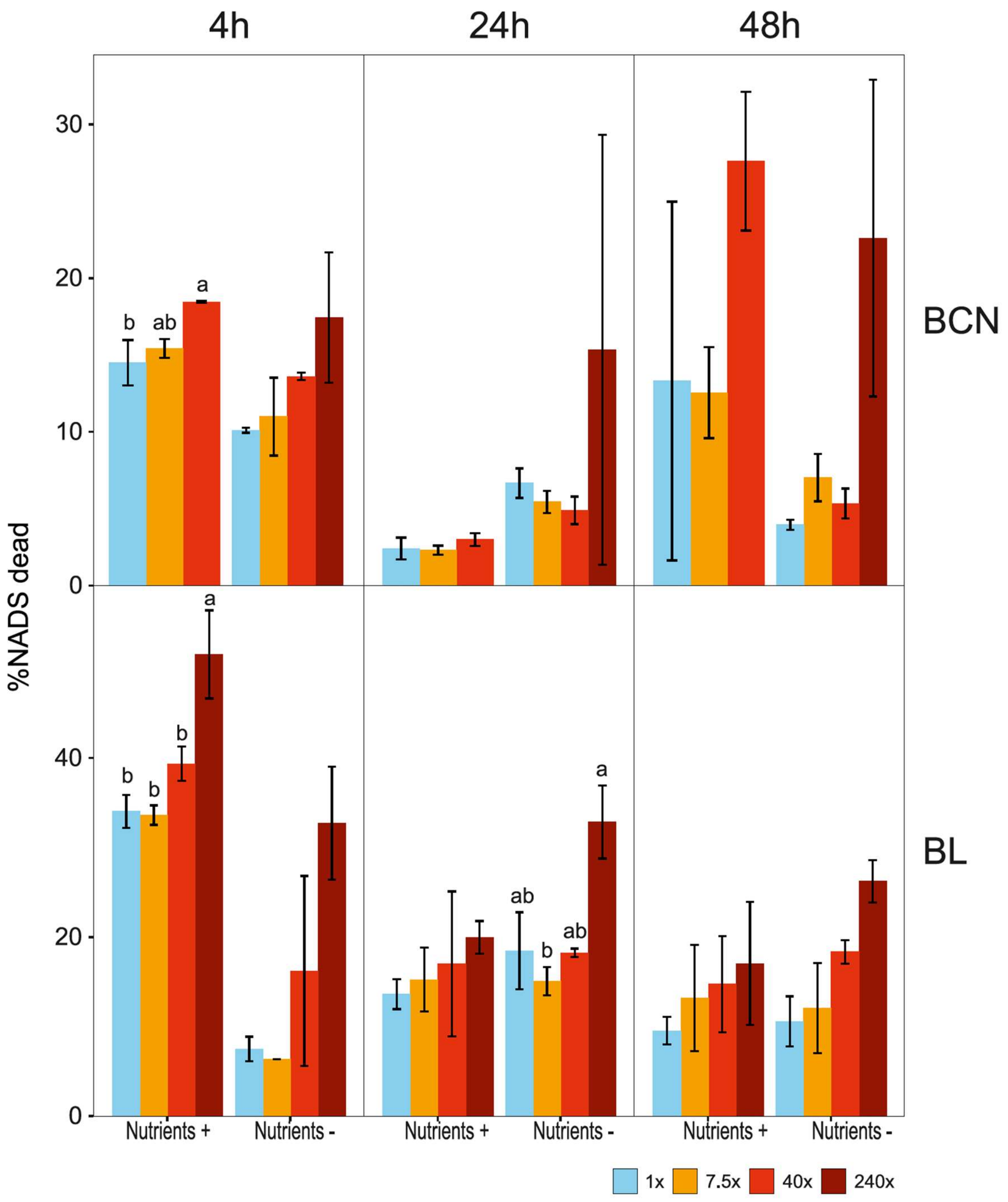

Figure S5. Percentages of damaged or dead cells (NADS-) in four different exposure concentrations of ADOC (1x, 7.5x, 40x and 240x of *in situ* concentrations) with and without nutrient additions in the Barcelona (BCN) and Blanes (BL) experiments. Values are means of duplicates. Significant differences ($p < 0.05$) of mean values from replicates were analyzed using 1-way ANOVA followed by a post-hoc Tukey's HSD test and labeled as different letters.

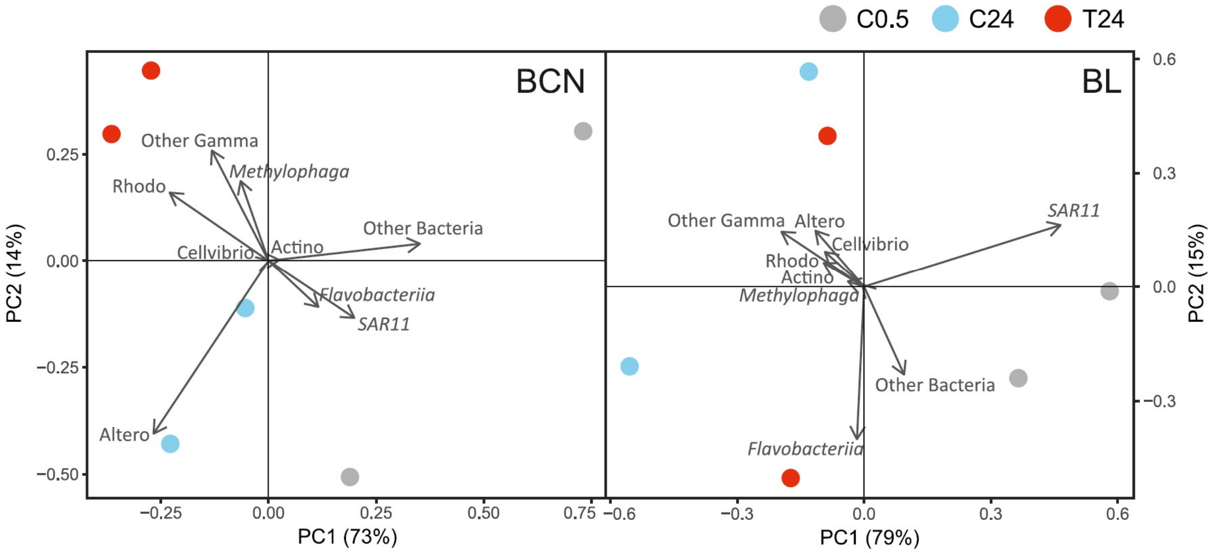

Figure S6. Principal component analysis (PCA) of the metagenomes in Barcelona (BCN) and Blanes (BL) using data from controls (C) and ADOC amendments (T) at initial time (0.5) and after 24 hours of ADOC exposure (24).

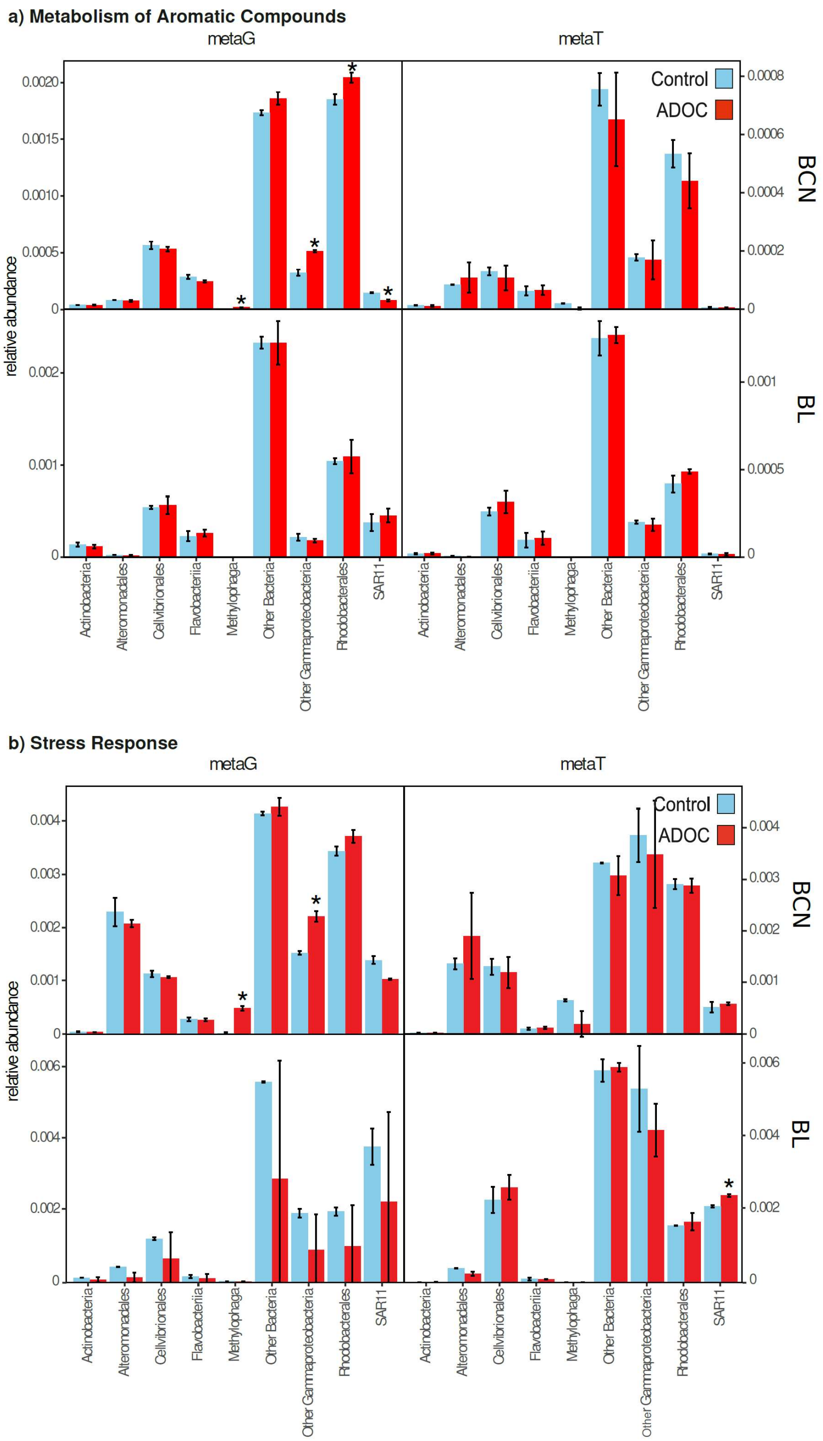

Figure S7. Relative abundance of genes grouped under SEED Metabolism of Aromatic compounds (top panel) and Stress Response (lower panel) in Barcelona (BCN) and Blanes (BL) metagenomes (metaG) after 24 h of ADOC exposure, in controls (C) and treatments (ADOC). Values are means of duplicates. Error bars show standard deviation. ADOC: ADOC amendment. Asterisks indicate significant differences between ADOC amendment and controls (t-test; $P < 0.05$).

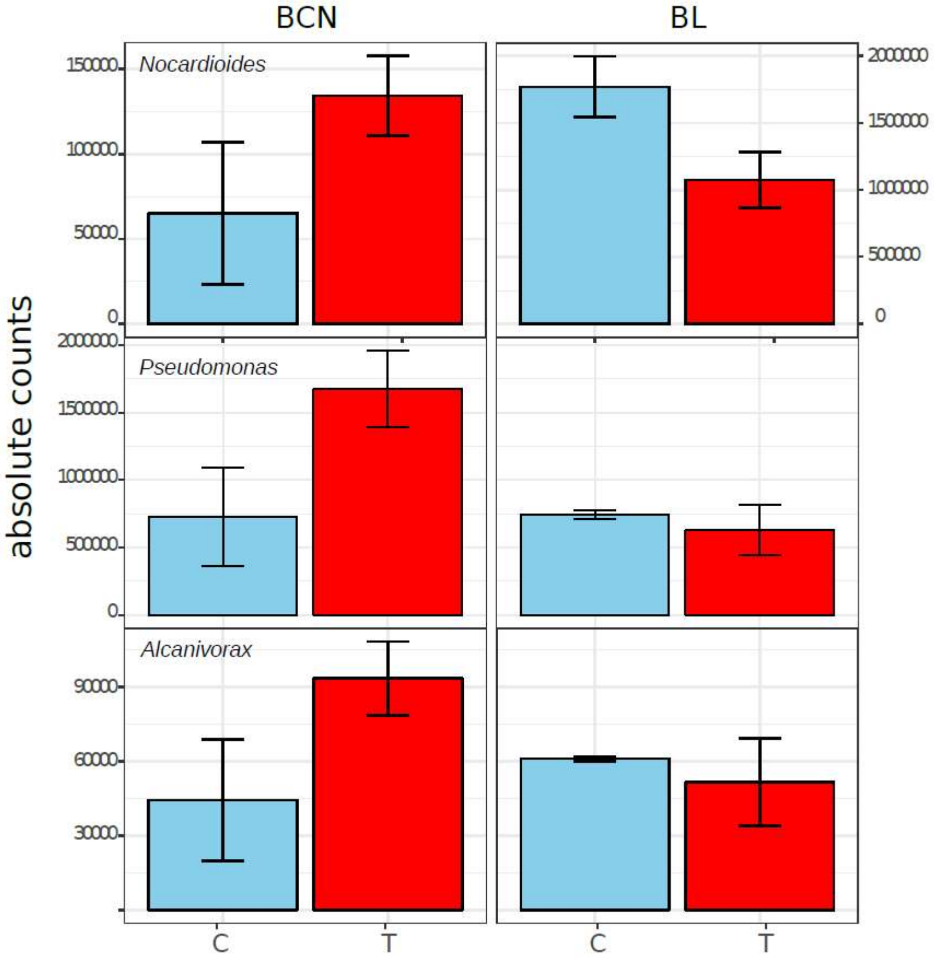

Figure S8. Absolute abundances of specific hydrocarbonoclastic bacteria (taken from Ghosal et al 2016) in Barcelona (BCN) and Blanes (BL) metagenomes (metaG) after 24 h of ADOC exposure, in controls (C) and ADOC treatments (T).
